# Review of the genus *Chasmogenus* Sharp, 1882 of northeastern South America with an emphasis on Venezuela, Suriname, and Guyana (Coleoptera, Hydrophilidae, Acidocerinae)

**DOI:** 10.3897/zookeys.934.49359

**Published:** 2020-05-19

**Authors:** Rachel R. Smith, Andrew Edward Z. Short

**Affiliations:** 1 Department of Ecology & Evolutionary Biology, and Division of Entomology, Biodiversity Institute, University of Kansas, Lawrence, KS 66045, USA University of Kansas Lawrence United States of America

**Keywords:** South America, aquatic beetles, new species, new synonymy, taxonomy

## Abstract

The water scavenger beetle genus *Chasmogenus* Sharp, 1882 is reviewed in northeastern South America using an integrative approach that combines adult morphology and molecular data from the gene cytochrome c oxidase I (COI). Eighteen new species are described: *Chasmogenus acuminatus***sp. nov.** (Brazil, French Guiana, Guyana, Suriname), *C. amplius***sp. nov.** (Venezuela), *C. berbicensis***sp. nov.** (Guyana), *C. brownsbergensis***sp. nov.** (Suriname), *C. castaneus***sp. nov.** (Venezuela), *C. clavijoi***sp. nov.** (Venezuela), *C. cuspifer***sp. nov.** (Venezuela), *C. flavomarginatus***sp. nov.** (Venezuela), *C. gato***sp. nov.** (Venezuela), *C. guianensis***sp. nov.** (Suriname, Guyana), *C. ignotus***sp. nov.** (Brazil), *C. ligulatus***sp. nov.** (Suriname), *C. lineatus***sp. nov.** (Venezuela), *C. pandus***sp. nov.** (Brazil, French Guiana, Suriname), *C. schmits***sp. nov.** (Suriname), *C. sinnamarensis***sp. nov.** (French Guiana), *C. tafelbergensis***sp. nov.** (Suriname), and *C. undulatus***sp. nov.** (Guyana). We found genetic support for an additional new species in Guyana which is currently only known from females that we refer to as *Chasmogenus* sp. *C.* We examined the holotypes of the four species previously known from the region, and found that *C. occidentalis* García syn. nov. and *C. yukparum* García syn. nov. are conspecific with *C. bariorum* García, 2000 and are synonymized with that species, which is here redescribed. We redescribe *C. australis* García and expand the range of this species to include northern Brazil, Guyana, and French Guiana. All species are aquatic, with most being associated with forested streams and forest pools. Of the 21 species, more than half (11) are only known from a single locality indicating the genus may have many more micro-endemic species yet to be discovered in the region. Characters of the male genitalia are essential for confirming the identity of some species, consequently it is not always possible to make positive identifications of unassociated female specimens based on morphology alone. Habitus images are provided as well as a revised key to the genus for northeastern South America.

## Introduction

The water scavenger beetle genus *Chasmogenus* Sharp (Hydrophilidae: Acidocerinae) is a widespread lineage that occurs in all regions except the Nearctic. Although the genus contains more than 45 described species worldwide, the Neotropical region has been poorly studied and contains only 15 described species (Clarkson and Ferreira-Jr 2014, Short 2005). Fieldwork over the last decade has generated thousands of new specimens of the genus from northern South America, particularly Venezuela, Suriname, and Guyana. Recent studies by García (2000; Venezuela) and Clarkson and Ferreira-Jr (2014; southeastern Brazil) have begun to elucidate the diversity of this genus in South America; however, it still remains largely unknown on the continent. This is partly due to the low degree of morphological variation between species, making identification and even routine morphospecies sorting a substantial challenge. Here, we take an integrative approach utilizing both adult morphology and molecular data from the mitochondrial gene cytochrome c oxidase I (COI) to review the genus for northeastern South America.

## Materials and methods

### Morphological methods

Morphological terminology largely follows Hansen (1991) except for the use of meso- and metaventrite instead of meso- and metasternum. All specimens were examined with an Olympus SZX7 (to 56× magnification) microscope. Measurements were taken with an ocular micrometer. Habitus photographs were taken with a Visionary Digital imaging system. All final images were created by stacking multiple individual photographs from different focal planes using the software Zerene Stacker. Males chosen for dissection were relaxed in 70% ethanol for at least eight hours and genitalia were cleared in warm 10% KOH solution in a water bath for approximately one hour. Cleared genitalia were placed and stored on slides in glycerin. Between 7–25 photos of genitalia were taken using an Olympus SZX16 (to 110×) and an Olympus BX51 (to 200×) and compiled via the focus stacking software CombineZP. We used Simplemappr (Shorthouse 2010) to create the distribution maps.

### Molecular methods

Total genomic DNA extractions were performed on whole beetles using a DNeasy tissue kit (Qiagen, Alameda, CA). Vouchers (Table 1) are deposited at the University of Kansas (Lawrence, KS, USA) unless otherwise indicated in the material examined section. We sequenced multiple populations of species that were widely distributed. We amplified the mitochondrial gene COI using the primers and protocols given in Short and Fikáček (2013). Amplification was successful for all species except for *C. castaneus* (for which an attempt to extract and amplify DNA from a pinned specimen failed) and *C. tafelbergensis* (which was a fresh specimen). We assembled and edited the resulting DNA sequences in Geneious 8.0.5 (Biomatters, http://www.geneious.com). We also used Geneious to calculate raw pairwise distances between sequences. All newly generated sequences are deposited in GenBank (Table 1). We used the IQ-Tree webserver (Nguyen et al. 2015) to conduct a maximum likelihood analysis on the COI data. The optimal model of substitution was selected using the Auto function in IQ-TREE. To assess nodal support, we performed 1000 ultrafast bootstrap replicates (Minh et al. 2013). We included the Central American species *Chasmogenus ruidus* Short, 2005 to root the tree (GenBank accession KC935240).

**Table 1.** List specimens and GenBank accession numbers used in this study.

Taxon	Voucher	Country: State/Site: Coordinates	Accession number
*C. acuminatus*	SLE445	Suriname: Kwamala: 2.175350, -56.787399	MT052762
	SLE516	French Guiana: Petit-Saut: 5.070, -53.029	MT052775
	SLE1081	Suriname: Raleighvallen: 4.681833, -56.185635	MT052770
	SLE1086	Guyana: Parabara: 2.108200, -59.227551	MT052760
	SLE1619	Suriname: Sipaliwini: 2.005700, -55.969151	MT052766
	SLE1623	Brazil: Para: -1.49292, -54.51566	MT052761
	SLE1804	Suriname: Kabalebo: 4.42313, -57.19198	MT052772
	SLE1805	Suriname: Kabalebo: 4.42313, -57.19198	MT052773
	SLE1820	Suriname: Kwamala: 2.182883, -56.787251	MT052765
	SLE1822	Suriname: Werehpai: 2.362933, -56.697681	MT052763
	SLE1823	Suriname: Werehpai: 2.362933, -56.697681	MT052764
	SLE1830	Suriname: Kappel: 3.791317, -56.149467	MT052767
	SLE1838	Suriname: Voltzberg: 4.673867, -56.184650	MT052769
	SLE1839	Suriname: Voltzberg: 4.673867, -56.184650	MT052768
	SLE1840	French Guiana: Petit-Saut: 5.09794, -53.06402	MT052776
	SLE1849	Suriname: Raleighvallen: 4.708000, -56.219318	MT052774
	SLE1850	Brazil: Amapá: 3.65822, -51.76958	MT052771
*C. amplius*	SLE1201	Venezuela: Amazonas: 4.980750, -67.739082	MT052788
*C. australis*	SLE1080	Venezuela: Barinas: 8.282567, -70.397781	MT052781
	SLE1082	Venezuela: Zulia: 10.043017, -71.007133	MT052782
	SLE1615	Guyana: Zilda Wao: 2.828733, -59.809101	MT052783
	SLE1621	Venezuela: Monagas: 9.096633, -62.726967	MT052780
	SLE1624	Brazil: Roraima: 0.730611, -60.432806	MT052784
	SLE1629	Brazil: Roraima: 3.305800, -60.857633	MT052779
	SLE1774	Brazil: Roraima: 1.58485, -61.001967	MT052778
	SLE1856	French Guiana: Yiyi: 5.419, -53.050	MT052777
*C. bariorum*	SLE078	Venezuela: Aragua: 10.373190, -67.742500	MT052749
	SLE530	Venezuela: Aragua: 10.373190, -67.742500	MT052747
	SLE531	Venezuela: Aragua: 10.373190, -67.742500	MT052748
	SLE534	Venezuela: Zulia: 9.841500, -72.821831	MT052745
	SLE1613	Venezuela: Guárico: 9.772017, -67.353348	MT052746
*C. berbicensis*	SLE1864	Guyana: Berbice: 4.146817, -58.237202	MT052787
*C. brownsbergensis*	SLE1828	Suriname: Brownsberg: 4.947850, -55.181850	MT052790
	SLE1861	Suriname: Brownsberg: 4.948900, -55.180416	MT052791
*C. clavijoi*	SLE1198	Venezuela: Guarico: 8.138267, -66.407654	MT052789
*C. cuspifer*	SLE532	Venezuela: Zulia: 10.373190, -67.742500	MT052750
	SLE533	Venezuela: Zulia: 9.841500, -72.821831	MT052751
*C. flavomarginatus*	SLE1083	Venezuela: Barinas: 8.807067, -70.518982	MT052740
	SLE1084	Venezuela: Tachira: 7.58396, -72.17233	MT052742
	SLE1235	Venezuela: Barinas: 8.807067, -70.518982	MT052741
*C. gato*	SLE1202	Venezuela: Amazonas: 4.980750, -67.739082	MT052785
*C. guianensis*	SLE1616	Guyana: Berbice: 4.154817, -58.178616	MT052792
	SLE1821	Suriname: Sipaliwini: 2.182883, -56.787251	MT052798
	SLE1826	Suriname: Palumeu: 2.477000, -55.629410	MT052800
	SLE1827	Suriname: Palumeu: 2.477000, -55.629410	MT052797
	SLE1834	Guyana: Berbice: 4.154817, -58.178616	MT052796
	SLE1835	Guyana: Berbice: 4.154817, -58.178616	MT052799
	SLE1836	Guyana: Berbice: 4.154817, -58.178616	MT052795
	SLE1862	Guyana: Berbice: 4.154817, -58.178616	MT052793
	SLE1863	Suriname: Palumeu: 2.477000, -55.629410	MT052794
*C. ignotus*	SLE1844	Brazil: Amazonas: -2.93079, -59.97514	MT052753
*C. ligulatus*	SLE474	Suriname: Sipaliwini: 2.977310, -55.384998	MT052759
*C. lineatus*	SLE1061	Venezuela: Lara: 10.1543, -69.9576	MT052743
	SLE1614	Venezuela: Guárico: 9.772017, -67.353348	MT052744
	SLE1772	Venezuela: Bum Bum: 8.300550, -70.753349	MT052742
*C. pandus*	SLE1858	Brazil: Amapa: 3.85039, -51.81683	MT052801
*C. schmits*	SLE1824	Suriname: Kutari: 2.175350, -56.787399	MT052786
*C. sinnamarensis*	SLE077	French Guiana: Petit-Saut: 5.070, -53.029	KC935241
	SLE517	French Guiana: Petit-Saut: 5.070, -53.029	MT052752
*C. undulatus*	SLE1618	Guyana: Region 8: 5.304350, -59.837616	MT052756
	SLE1831	Guyana: Region 8: 5.304350, -59.837616	MT052755
	SLE1832	Guyana: Region 8: 5.304350, -59.837616	MT052754
	SLE1833	Guyana: Region 8: 5.304350, -59.837616	MT052757
*C.* sp. C	SLE1783	Guyana: Region 6: 4.146817, -58.237202	MT052758

### Depositories of examined material

**CBDG** Center for Biological Diversity, University of Guyana, Georgetown

**INPA**Instituto Nacional de Pesquisas da Amazonia, Colecao Sistematica da Entomologia, Manaus, Brazil (N. Hamada))

**MALUZ** Museo de Artrópodos de la Universidad del Zulia, Maracaibo, Venezuela (J. Camacho, M. García)

**MIZA** Museo del Instituto de Zoologia Agricola, Maracay, Venezuela (L. Joly)

**NZCS** National Zoological Collection of Suriname, Paramaribo (A. Gangadin, V. Kadosoe)

**SEMC**Snow Entomological Collection, University of Kansas, Lawrence, KS (A. Short))

**USNM** U.S. National Museum of Natural History, Smithsonian Institution, Washington, DC U.S. National Museum of Natural History, Smithsonian Institution, Washington, DC (C. Micheli)).

## Results

**Figure 1.**
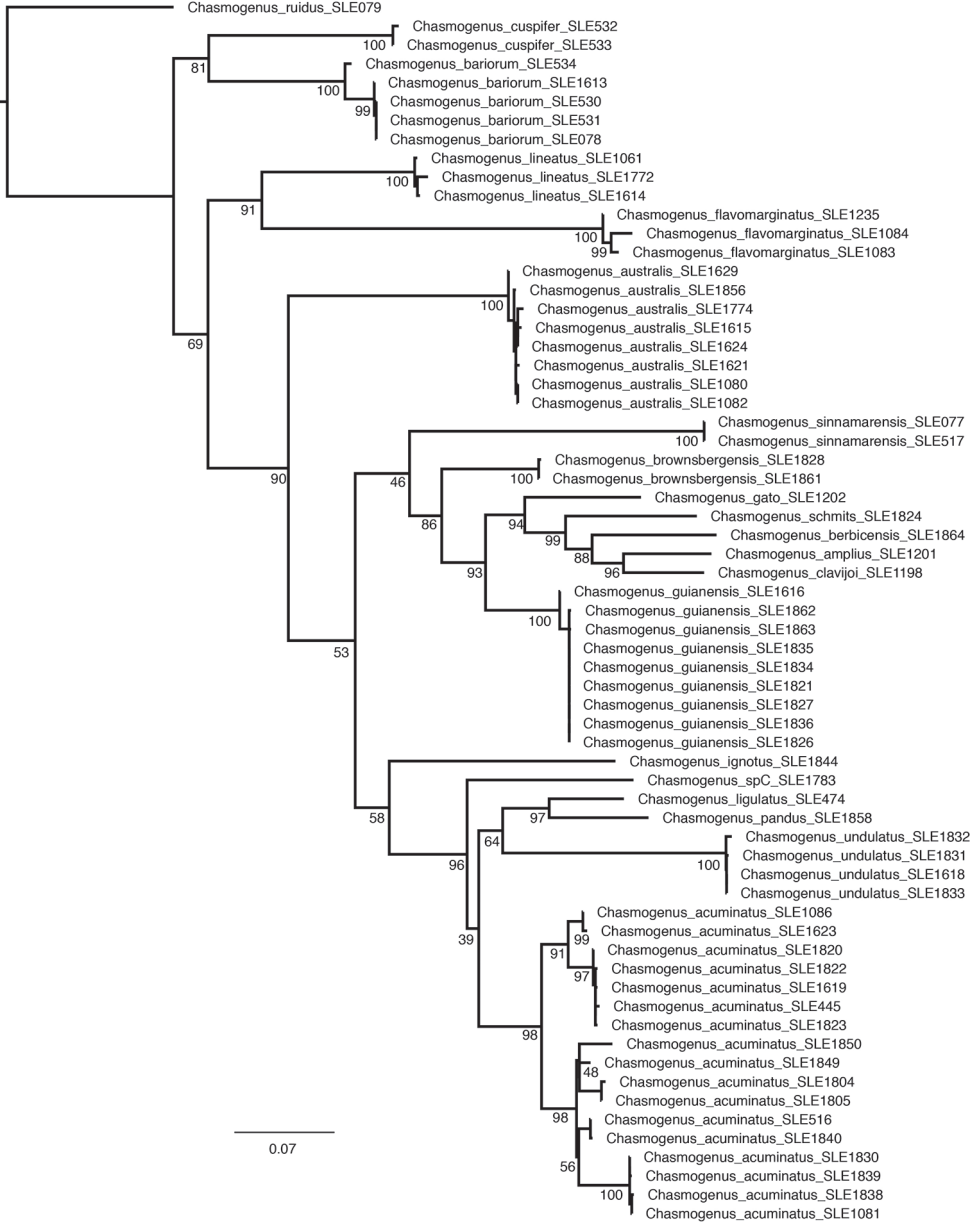
Maximum likelihood phylogeny of *Chasmogenus* spp. based on COI sequences. Ultra Fast Bootstrap (UFBS) values are indicated at nodes.

The results of the Maximum Likelihood analysis (Fig. 1) found all morphological species recognized here to be reciprocally monophyletic. The smallest interspecific raw divergence between any species pair was 7.1% (between *C. pandus* and *C. ligulatus*) followed by 7.2% (between *C. amplius* and *C. clavijoi*). The sequence divergence between all other species was greater than 8.0%. The maximum intraspecific raw sequence divergence was 2.1% (most were below 1.0%) with one exception: the distance between individuals of *C. acuminatus* was as high as 6.0%, which we discuss further below. A table showing the pairwise genetic distances between all individuals is shown in Suppl. material 1: Table S1.

### List of species

1. *Chasmogenus acuminatus* sp. nov. Brazil (Amapá, Pará), French Guiana, Guyana, Suriname

2. *Chasmogenus amplius* sp. nov. Venezuela

3. *Chasmogenus australis* García, 2000 Venezuela, French Guiana, Guyana, Brazil (Roraima)

4. *Chasmogenus bariorum* García, 2000 Venezuela

*Chasmogenus occidentalis* García, 2000 syn. nov.

*Chasmogenus yukparum* García, 2000 syn. nov.

5. *Chasmogenus berbicensis* sp. nov. Guyana

6. *Chasmogenus brownsbergensis* sp. nov. Suriname

7. *Chasmogenus castaneus* sp. nov. Venezuela

8. *Chasmogenus clavijoi* sp. nov. Venezuela

9. *Chasmogenus cuspifer* sp. nov. Venezuela

10. *Chasmogenus flavomarginatus* sp. nov. Venezuela

11. *Chasmogenus gato* sp. nov. Venezuela

12. *Chasmogenus guianensis* sp. nov. Suriname, Guyana

13. *Chasmogenus ignotus* sp. nov. Brazil (Amazonas)

14. *Chasmogenus ligulatus* sp. nov. Suriname

15. *Chasmogenus lineatus* sp. nov. Venezuela

16. *Chasmogenus pandus* sp. nov. Brazil (Amapá), French Guiana, Suriname

17. *Chasmogenus schmits* sp. nov. Suriname

18. *Chasmogenus sinnamarensis* sp. nov. French Guiana

19. *Chasmogenus tafelbergensis* sp. nov. Suriname

20. *Chasmogenus undulatus* sp. nov. Guyana

21. *Chasmogenus* sp. C Guyana

### Characters of taxonomic importance

Species of New World *Chasmogenus* are generally quite similar morphologically. There are several characters that easily separate species into rough species-groups, such as the condition of the labro-clypeal emargination, the elevation of the mesoventrite, and general size. However, within each of these groups, the aedeagus is often the only diagnostic feature. For a number of species, especially in the Amazon region, unassociated females cannot be identified with confidence using morphology alone. Here we review some characters that show inter- and/or intraspecific variation.

**Figure 2.**
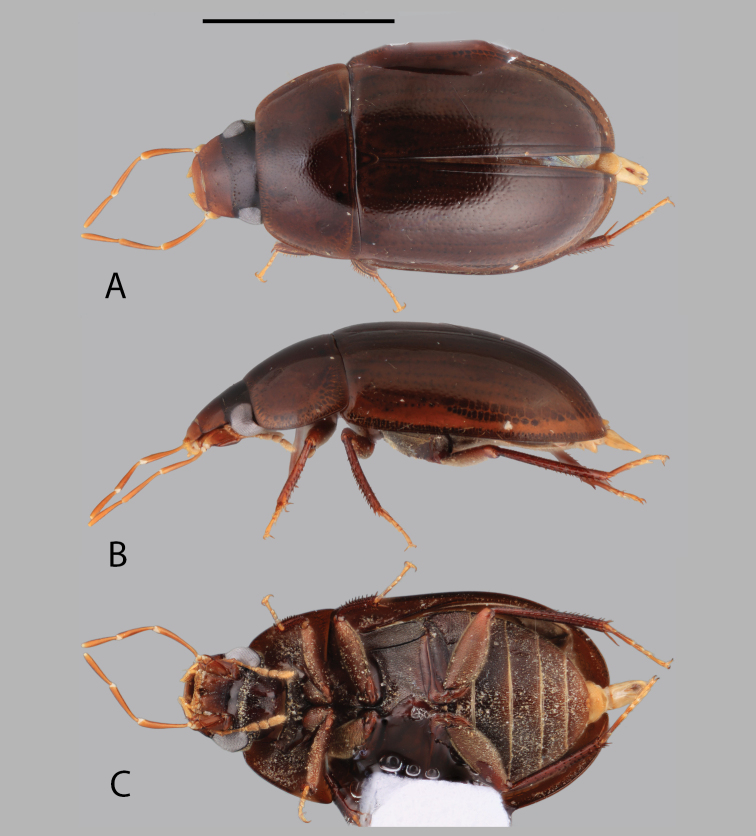
Habitus of *Chasmogenus amplius*: **A** dorsal view **B** lateral view **C** ventral view. Scale bar: 2 mm.

**Figure 3.**
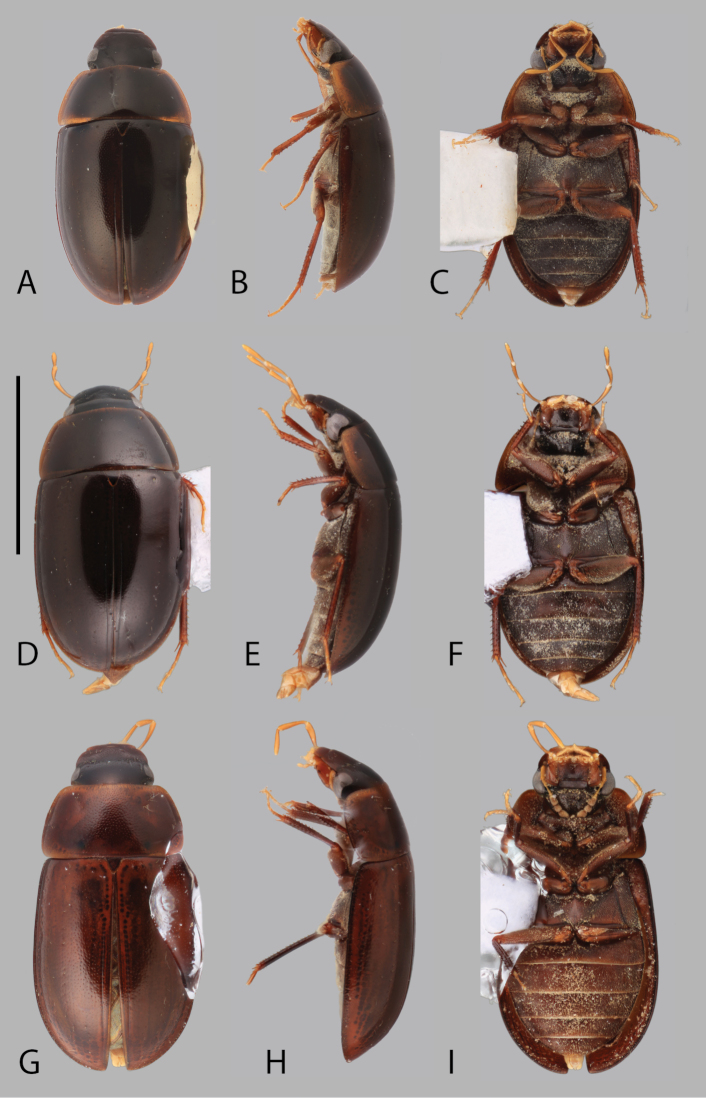
Habitus of *Chasmogenus* spp. **A–C***C. flavomarginatus*: **A** dorsal view **B** lateral view **C** ventral view. **D–F***C. bariorum*: **D** dorsal view **E** lateral view **F** ventral view. **G–I***C. castaneus***G** dorsal view **H** lateral view **I** ventral view. Scale bar: 2 mm.

**Figure 4.**
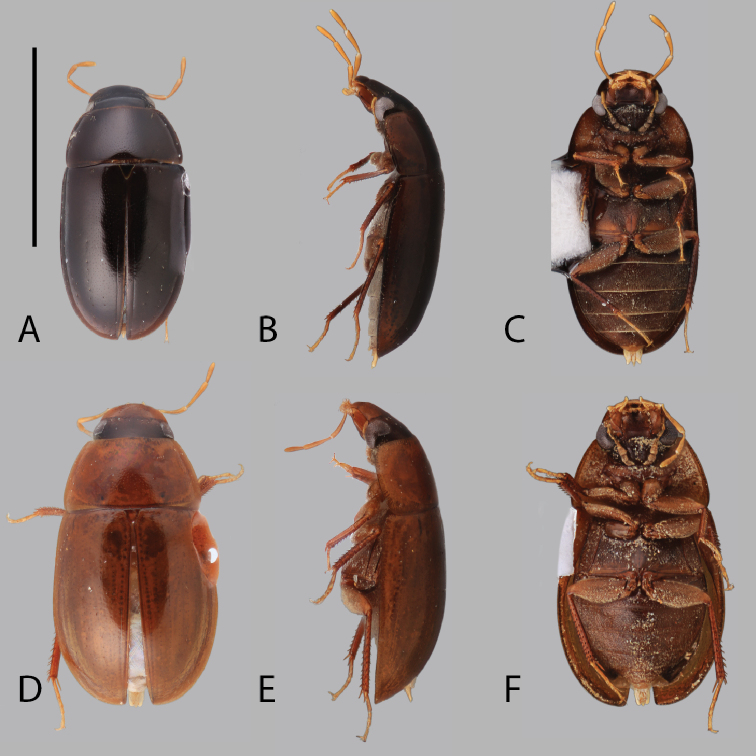
Habitus of *Chasmogenus* spp. **A–C***C. lineatus*: **A** dorsal view **B** lateral view **C** ventral view. **D–F***C. australis*: **D** dorsal view **E** lateral view **F** ventral view. Scale bar: 2 mm.

**Figure 5.**
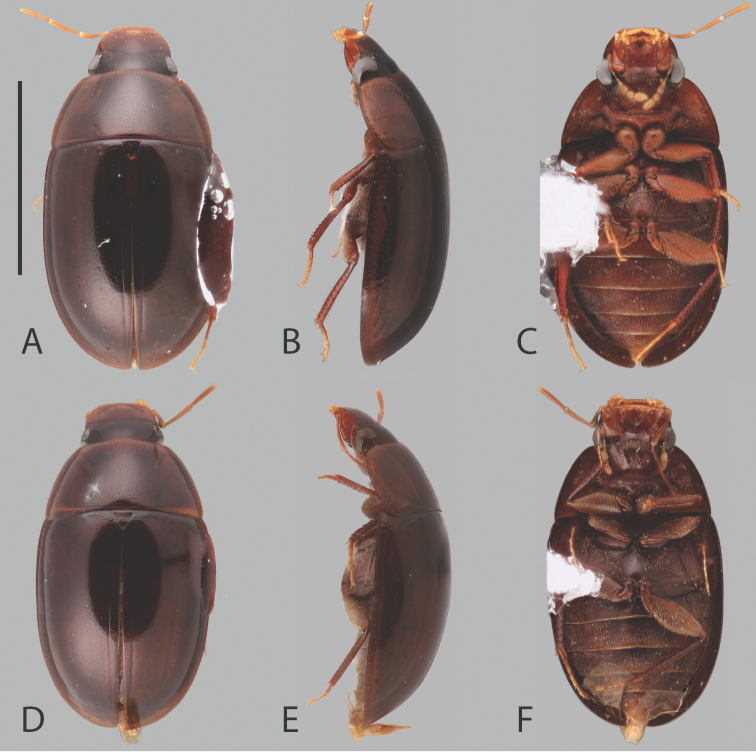
Habitus of *Chasmogenus* spp. **A–C***C. acuminatus*: **A** dorsal view **B** lateral view **C** ventral view. **D–F***C. pandus*: **D** dorsal view **E** lateral view **F** ventral view. Scale bar: 2 mm.

**Dorsal coloration.** The dorsal coloration of most species ranges from shades of brown to dark red-brown (Figs 2A–C, 3A–I, 4A–F, 5A–F), but in a few cases (e.g., *C. australis*, Fig. 4D–F) may be much paler and appear almost yellow. Care should be taken not to confuse pale teneral specimens with true pale coloration. The coloration of the dorsum of the head is useful in diagnosing a few species, as some have uniformly colored heads (e.g., *C. lineatus*, Fig. 8F) and others may be bicolored (e.g., *C. australis* and *C. amplius*, Figs 8B, 9A), or appearing translucent (e.g., *C. pandus* Fig. 10D). Distinct pale preocular patches may be found on only one species (e.g., *C. flavomarginatus*, Fig. 8E). Most species for which long series are available exhibit some intraspecific dorsal color variation and this character should not be used alone for definitive identification.

**Labro-clypeal margin.** The anterior margin of the clypeus is a key feature for separating groups of species. In most species, the clypeus emarginates anteroposteriorly exposing a gap between the clypeus and labrum, however in the absence of this gap in which the clypeus and labrum are contiguous, it is diagnostic of some species (e.g., *C. flavomarginatus*, and *C. lineatus*, Fig. 8E, F). The shape of the gap and the degree to which the clypeus emarginates is generally uniform within species, and is helpful in determining species identification, but is not a unique diagnostic feature of any species.

**Mentum.** In all species, the anterior half to two-thirds of the mentum is depressed to a varying degree with a medial notch of variable shape, usually either triangular or rounded. The depression of the mentum may or may not be interrupted with a low curved ridge situated just posterior to the medial notch, a feature that is found in several species (e.g., *C. lineatus*, *C. cuspifer*, and *C. sinnamarensis*) and may serve to distinguish these from other closely related taxa.

**Mesoventrite.** The condition of the mesoventrite is markedly variable in elevation; though always forming a longitudinal carina, it varies from a very low, faint carina to a distinctly raised acute tooth. When it is raised into a tooth, it is diagnostic of either *C. bariorum* or *C. cuspifer*; form of the tooth is slightly variable, sometimes rounded on the posterior slope to linear on the anterior and posterior slope in an acute triangular form (e.g., Fig. 7A, B). In many other species, it is indistinct and very weakly elevated, in others with a mild elevation, it may be convex along the outer margins.

**Aedeagus.** The aedeagus is the primary and sometimes only definitive diagnostic feature for a number of the species included here. Additionally, we found substantially more variation in form of the genitalia than had previously been reported for the genus. Most species have a rather “normal” trilobed form with the relative length and shape of the parameres and median lobe being the most helpful in separating species. One group of species (e.g., *C. acuminatus*, *C. undulatus*, *C. ligulatus*, *C. pandus*, Fig. 15A–E, G–I) possess a large and often asymmetrical sclerite associated with the median lobe, the presence and shape of this sclerite is important to separating species. Two species exhibit a strikingly modified and asymmetrical aedeagus (*C. ignotus* and *C. tafelbergensis*, Fig. 16). In these two species the parameres are greatly enlarged, with one wider than the other, and the median lobe is reduced to a narrow strip. In addition, the aedeagus is very deep and three dimensional.

### Taxonomic treatment

#### Chasmogenus

Taxon classificationAnimaliaColeopteraHydrophilidae

Sharp, 1882

428E4283-A72B-5A8B-A84F-D71E8A2EB479

Chasmogenus Sharp, 1882: 73.

##### Differential diagnosis.

Moderately sized beetles, elongate oval in dorsal view, moderately dorsoventrally compressed, 2.5–6.2 mm in total length. Dorsal coloration from light tan-yellow to very dark brown. Ground punctation of the head fine to moderately coarse, labrum with slightly finer ground punctation and almost indistinguishably small systematic punctures that bear long golden setae along the distoanterior margin. Anterior margin of labrum slightly concave medially. Systematic punctures present on the clypeus, pronotum, and elytra. Elytra with very fine to coarse ground punctation and five loosely organized longitudinal rows of sparsely setose systematic punctures, sometimes only distinguishable by presence of setae; with sharply impressed sutural striae. Antennae with eight (Neotropical species) or nine antennomeres (Old World species). Maxillary palps half as long to longer than width of head posterior to eyes. Anterior half of mentum depressed with anteromedial notch, notch variable from shallowly rounded to triangular. Prosternum pubescent, form variable from evenly smooth to moderately tectiform. Mesoventrite either weakly elevated into medial longitudinal carina, or strongly elevated into posteroapical tooth. Metaventrite with median ovoid glabrous region, extending ca. half to slightly more than half the length of the metaventrite and ca. twice as long as it is wide. Metafemora densely and uniformly pubescent basally with a distal glabrous region of variable proportion. Abdomen densely and uniformly pubescent with golden to light yellow setae. Fifth abdominal ventrite with rounded posteromedial emargination lined with short, bristle-like setae. Size and form of aedeagus variable.

#### Chasmogenus acuminatus sp. nov.

Taxon classificationAnimaliaColeopteraHydrophilidae

B5B28386-542C-5147-97EE-8D342E7E8EAA

http://zoobank.org/CBA0DEFA-D4FD-4066-80A4-E4187B8B41D2

Figures 5A–C , 9B , 15H–I , 18 , 21E , 22C, D

Chasmogenus sp. X Short, 2013: 87 (in part); Short & Kadosoe, 2011: 87 (in part), Short, Salisbury, & La Cruz 2018: 193 (in part).

##### Type material.

**Holotype (male)**: “Suriname: Sipaliwini District/2°21.776'N, 56°41.861'W, 237 m/ Camp 3 Wehepai; leg. Short &/ Kadosoe; sandy forest creek/4–6.ix.2010; SR10-0904-01A/ CI-RAP Survey”, “DNA VOUCHER/ Extraction #/ SLE-1822”, “HOLOTYPE/ CHASMOGENUS/ acuminatus sp. n./ des. Smith & Short.” (NZCS). **Paratypes (321): Brazil: Amapa**: Oiapoque (c. 22 km S) on BR-156, leg. Short, forested detrital pools, BR18-0720-01B (1 ex., SEMC, DNA voucher SLE1850). **Para**: Vale do Paraíso, ca. 55 km N. Alenquer, -1.49292, -54.51566, leg. Short, detrital pool, BR18-0203-01B (1 ex., SEMC, DNA voucher SLE1623). **French Guiana**: Petit-Saut, 5.070, -53.029 (2 exs., SEMC, DNA Vouchers SLE516 and SLE1840). **Guyana: Region 9**: along road to Parabara, 2°09.557'N, 59°17.569'W, 268 m, 1.xi.2013, leg. Short, Isaacs and Salisbury, forest pools near Mushai Wao, GY13-1101-02A (4 exs., SEMC); Parabara, trail to mines, 2°05.095'N, 59°14.174'W, 250 m, 2.xi.2013, leg. Short, Isaacs and Salisbury, detrital pools in forest, GY13-1102-01A (2 exs., SEMC); North of Parabara, Bototo Wau Creek, 2°10.908'N, 59°20.306'W, 289 m, 31.x.2013, leg. Short, Isaacs and Salisbury, stream margins, GY13-1031-01A (3 exs., SEMC); Parabara north side of river, 2°06.492'N, 59°13.653'W, 274 m, 3.xi.2013, detritus margins and leaf packs, GY13-1103-02A (2 exs., SEMC, including DNA Voucher SLE1086). **Region 6**: Upper Berbice circa 1 km south of Basecamp 1, 4°09.241'N, 58°10.627'W, 109 m, 25.ix.2014, leg. Short and La Cruz, margins of creek with leaf packs and mud, GY14-0925-01B (2 exs., SEMC); Upper Berbice Basecamp 1, 4°09.289'N, 58°10.717'W, 96 m, 21.ix.2014, leg, Short, Salisbury and La Cruz, muddy detrital pools in drying creekbed near camp, GY14-0921-02A (5 exs., SEMC, CBDG); same data as previous except: 24.ix.2014, margins of basecamp creek, GY14-0924-01A (1 ex., SEMC); Upper Berbice circa 1 km west of Basecamp 1, 4°09.143'N, 58°11.207'W, 105 m, 22.iv.2014, leg, Short, Salisbury and La Cruz, margins of creek, GY14-0921-03H (2 exs., SEMC); same data as previous except: 21.ix.2014, leg. A. Short, sandy stream, GY14-0921-03A (1 ex. SEMC); Upper Berbice Basecamp 2, 4°45.301'N, 58°00.404'W, 49 m, 26.ix.2014, leg. Short, Salisbury and La Cruz, shallow detrital pool in forest draining into creek, GY14-0926-01A (2 exs. SEMC). **Region 8**: Konawaruk River, Basecamp 2 (NARIL basecamp), 5°07.539'N, 59°06.732'W, 80 m, 15.ix.2014, leg. Salisbury and La Cruz, unnamed clear water creek, slow flowing and shallow, GY14-0915-02 (7 exs., SEMC); Upper Potaro Camp (circa 7 km northwest of Chenapau), 5°0.660'N, 59°38.283'W, 484 m, 11.iii.2014, leg. Short, Baca, Salisbury and La Cruz, Potaro margin trail with wet detritus in sandy area, GY14-0311-04A (1 ex., SEMC). **Region 10**: Upper Berbice logging road KM 1, 5°03.892'N, 58°03.303'W, 71 m, 29.ix.2014, leg. Short, Salisbury and La Cruz, marsh and creek, GY14-0929-01B (1 ex., SEMC). **Suriname: Sipaliwini District**: Camp 3, Werehpai, 2°21.776'N, 56°41.861'W, 237 m, 3–7.ix.2010, leg. Short and Kadosoe, detrital pools forest, 2010 CI-RAP Survey, SR10-0903-02A (4 exs., SEMC); same data as previous except: 3.ix.2010, pooled up detrital creek, 2010 CI-RAP Survey, SR10-0903-01A (54 exs., NZCS, SEMC, INPA); same data as previous except: 4–6.ix.2010, sandy forest creek, SR10-0904-01A (26 exs., SEMC, including DNA vouchers SLE1822 and SLE1823); Camp 2 on Sipaliwini River 2°10.973'N, 56°47.235'W; 210 m, 28.viii.2010, Short and Kadosoe, small detrital stream, CI-RAP Survey, SR10-0828-03A (27 exs., SEMC); same data as previous except: 30.viii.2010, forest creek, SR10-0831-01A (24 exs., SEMC); same data as previous except: 31.viii.2010, sandy forest creek with detritus, SR10-0831-01B (38 exs., SEMC); same data as previous except: 28–29.viii.2010, large forest stream, SR10-0828-02A (16 exs., SEMC, including DNA voucher SLE1820); Camp 1 on Kutari River, 2°10.521'N, 56°47.244'W, 228 m, 20.viii.2010, leg. Short and Kadosoe, forest stream, CI-RAP Survey, SR10-0820-01A (7 exs., SEMC); same data as previous except: 19.viii.2010, SR10-0819-02A (1 ex., SEMC); same data as previous except: 19.viii.2010, forested swamp, SR10-0819-01A (39 exs., SEMC); same data as previous except: 19–24.viii.2010, leg. Short, Kadosoe, and Larsen, FIT, SR10-0819-TN1 (1 ex., SEMC); rapids on Kutari River, 2°19.280'N, 56°52.595'W, 224 m, 18.viii.2010, leg. A. E. Z. Short, forest stream, 2010 CI-RAP Survey, SR10-0818-01A (1 ex., SEMC); Camp 2 on Sipaliwini River, 2°10.973'N, 56°47.235'W, 210 m, 29–30.viii.2010, leg. Short and Kadosoe, inselberg, 2010 CI-RAP Survey, SR10-0829-01A (1 ex. SEMC); Camp 1 on Kutari River, 2°10.521'N, 56°47.244'W, 228 m, 22.viii.2010, Short and Kadosoe, forest swamp, CI-RAP Survey, SR10-0822-02A (7 exs., SEMC including DNA Voucher SLE 445); Camp 1 Upper Palumeu, 2.47700N, 55.62941W, 275 m, 10–16.iii.2012, leg. A. Short, 2012 CI-RAP Survey, Flight Intercept Trap, SR12-0310-TN1 (1 ex. SEMC); Raleighvallen Nature Reserve, base of Voltzberg, 4°40.432'N, 56°11.079'W, 86 m, 16.iii.2016, leg. Short et al., pooled up stream, SR16-0316-01B (10 exs., SEMC, including DNA vouchers SLE1838 and SLE1839); Raleighvallen Nature Reserve Lolopaise area, 4°42.48'N, 56°13.15908'W, 24 m, 18.iii.2016, leg. Short et al., intermittent stream margins and flotation, SR16-0318-01D (3 exs., SEMC, including DNA voucher SLE1849); Raleighvallen Nature Reserve, trail from plateau to Voltzberg, 17.iii.2016, leg. J. Girón, stream with roots and mud, SR16-0317-04A (3 exs., SEMC); Raleighvallen Nature Reserve, Fungu Island, 4°43.459'N, 56°12.658'W, 30 m, 14.iii.2016, leg. A. Short, isolated river margin pools with rocky bottom, SR16-0314-01E (1 ex., SEMC); Raleighvallen Nature Reserve Voltzberg Station, 04°40.910'N, 56°11.138'W, 78 m, 29.vii.2012, leg. A. Short and C. McIntosh, detrital side pool, SR12-0729-02B (2 exs., SEMC); same data as previous except: 29.vii.2012, leg. Short, Maier, McIntosh, and Kadosoe, stream margins, SR12-0729-02A (3 exs., SEMC); Raleighvallen Nature Reserve, trail to Raleighvallen, 04°42.480'N, 56°13.159'W, 24 m, 27.vii.2012, leg. C. McIntosh, detrital pools near creek in forest, SR12-0727-03D (5 exs., SEMC); Raleighvallen Nature Reserve Voltzberg trail, 04°40.910'N, 56°11.138'W, 78 m, 30.vii.2012, leg. A. Short and C. McIntosh, detrital pools along stream, SR12-0730-01B (6 exs., SEMC, including DNA Voucher SLE1081); CSNR Tafelberg Summit near Augustus Creek Camp, 3°55.600'N, 56°11.300'W, 600 m, 16.viii.2013, leg. Short and Bloom, pond on trail into Arrowhead basin, SR13-0816-02A (1 ex., SEMC); same data as previous except: 22.viii.2013, detrital creek, SR13-0822-01A (1 ex., SEMC); Sipaliwini Savanna Nature Reserve, Four Brothers Mountain, 2°00'20.5"N, 55°58'08.9"W, 337 m, 31.iii.2017, leg. A. Short, detrital pools, SR17-0331-01D (1 ex., SEMC DNA Voucher SLE1619); Kabalebo Nature Resort, Moi Moi Creek, leg. Short, detrital pool, SR19-0310-01G (2 exs., SEMC, DNA vouchers SLE1804 and SLE1805).

##### Differential diagnosis.

Among species that have a broad clypeal emargination and the apex of the median lobe extending past the apex of the parameres, *C. acuminatus* may be distinguished by the straight outer margin (Fig. 15H, I) of the parameres, (distinctly sinuated in the similar and regionally co-occurring *C. undulatus*, *C. ligulatus*, and *C. pandus*). Examination of the aedeagus is the only way to definitively identify this species. Unassociated females may not be determined with certainty.

##### Description.

***Size and color.*** Total body length 3.4–3.9 mm. Body form elongate oval with slightly curved lateral margins. Dorsum of head brown to dark brown, clypeus distinctly paler brown (Fig. 9B). Pronotum and elytra uniformly dark orange-brown (Fig. 5A). Venter dark orange centrally, dark red-brown distally (Fig. 5C). ***Head.*** Ground punctation on head coarse. Clypeus with anteromedial emargination, which exposes a broadly rounded to angulate gap between the clypeus and labrum (Fig. 9B). Mentum strongly depressed in anterior half with a triangular to subtriangular anteromedial notch. Maxillary palps long, longer than width of head immediately posterior to eyes. ***Thorax.*** Ground punctation on pronotum moderately coarse (Fig. 5A). Prosternum tectiform. Mesoventrite with weak elevation forming a thin posteromedial longitudinal carina. Metafemora densely pubescent in basal nine-tenths (Fig. 5C). ***Aedeagus.*** Aedeagus (Fig. 15H, I) with median lobe subtriangular in shape, widest at base and gradually tapering along entire length; apex acute, distinctly extending beyond the apex of the parameres. Sclerite of the median lobe expanded, but very narrow and weakly developed, apex not reaching the apex of the parameres. Gonopore situated ca. one gonopore width below the apex of the median lobe. Parameres symmetrical, with outer margins very slightly inwardly curved a long length; width of each paramere gradually narrowed to a blunt (Fig. 15H) to slightly acute (Fig. 15I) apex. Basal piece short, ca. one-third the length of the parameres.

##### Etymology.

The species name is derived from the Latin *acuminatus*, meaning “pointed”, after the condition of the aedeagus, in which the apex of the median lobe is extended and forms an acute point.

##### Distribution.

Known from a broad range in the eastern Guiana Shield region of South America, from Guyana east to the state of Amapá, Brazil and south to the Amazon River (Fig. 18).

##### Biology.

This species is relatively widespread and one of the most commonly encountered *Chasmogenus* in the eastern Guiana Shield. It is found in forested habitats, typically associated with detrital pools, the margins of streams and creeks, and forested swamps (Figs 21E, 22C, D). Some specimens have been collected in flight intercept traps (FITs).

##### Remarks.

There is a high level of genetic diversity in the species (Fig. 1), with observed COI divergence as high as 6% between some individuals. However, we did not identify any substantial corresponding morphological variation and believe it is best to consider this intraspecific genetic variation for the time being.

#### Chasmogenus amplius sp. nov.

Taxon classificationAnimaliaColeopteraHydrophilidae

395EBE42-87AA-5225-910E-16867602E65A

http://zoobank.org/C5534EEE-91ED-4EE6-94AA-4BC0C181826E

Figures 2A–C , 9A , 14A , 17 , 20A

##### Type material.

**Holotype (male)**: “Venezuela: Amazonas State/ 4°58.838'N, 67°44.341'W; 95m/ Communidad Caño Gato, on Rio/ Sipapo; 16.i.2009; leg. Short/ Miller, Camacho, Joly, & García/ VZ09-0116-01X: along stream”, “[barcode]/ SM0843452/ KUNHM-ENT”, “HOLOTYPE/ CHASMOGENUS/ amplius sp. n./ des. Smith & Short” (MIZA). **Paratypes (57): Venezuela: Amazonas**: Same data as holotype (53 exs., MIZA, SEMC, including DNA voucher SLE1201); stream along Rio Sipapo, 4°55.849'N, 67°44.645'W, 87 m, 16.i.2009, leg. Short, García, Camacho, Miller and Joly, stream habitats, VZ09-0116-02X (2 exs., SEMC); Communidad Caño Gato, 4°58.845'N, 67°44.345'W, 100 m, 7.i.2006, leg. A.E.Z. Short, stream margin/detritus, AS-06-016 (2 exs., SEMC).

##### Differential diagnosis.

The large size (ca. 5.0 mm) and broad body form (Fig. 2A–C) of this species serve to differentiate *C. amplius* from most other congeners, and no other sympatric species may be confused with it. It is approximately the same size as and is morphologically similar to *C. berbicensis* from eastern Guyana, but may be distinguished by its paler dorsal coloration.

##### Description.

***Size and color.*** Total body length 4.5–5.0 mm. Body form elongate oval with broad, slightly curved lateral margins. Dorsum of head bicolored, frons dark brown, clypeus and labrum distinctly paler (Fig. 9A). Pronotum and elytra dark orange-brown to dark brown (Fig. 2A). Venter dark red-brown centrally, dark brown marginally (Fig. 2C). Abdominal ventrites orange-brown. ***Head.*** Ground punctation on head fine. Clypeus with anteromedial emargination, which exposes a rounded to angulate gap between clypeus and labrum (Fig. 9A). Mentum strongly depressed on anterior half with subtriangular anteromedial notch. Maxillary palps long, longer than width of head immediately posterior to eyes. ***Thorax.*** Ground punctation on pronotum fine. Prosternum weakly tectiform. Mesoventrite with weak elevation forming a posteromedial longitudinal carina. Metafemora densely pubescent in basal nine-tenths (Fig. 2C). ***Aedeagus.*** Aedeagus (Fig. 14A) with median lobe widest at base and very slightly tapering until the apical quarter, then narrowing abruptly to form an acute triangular apex which is even with or slightly extends beyond the apex of the parameres. Sclerite of the median lobe not expanded. Gonopore situated less than half of one gonopore width below the apex of the median lobe. Parameres symmetrical, with outer margins straight, with apex slightly inwardly curved and bluntly rounded. Basal piece long, ca. three-quarters the length of the parameres.

##### Variation.

There is a fair amount of variation in dorsal coloration; most specimens examined are medium-orange brown, whereas some are slightly paler.

##### Etymology.

The species name is derived from the Latin *amplius*, meaning “larger”, after the large body size of this species, the largest known species of the genus in northern South America.

##### Distribution.

Known from a few closely situated localities along the Orinoco River in southern Venezuela (Amazonas) (Fig. 17).

##### Biology.

This species has been collected in series along the margins of a small sandy stream that flowed into the Rio Sipapo (Fig. 20A). Specimens were found by agitating marginal areas where leaf detritus and sand had accumulated.

#### Chasmogenus australis

Taxon classificationAnimaliaColeopteraHydrophilidae

García, 2000

790D65B5-C650-57C9-A8DA-37CBE1C77EB9

**Figure 6.**
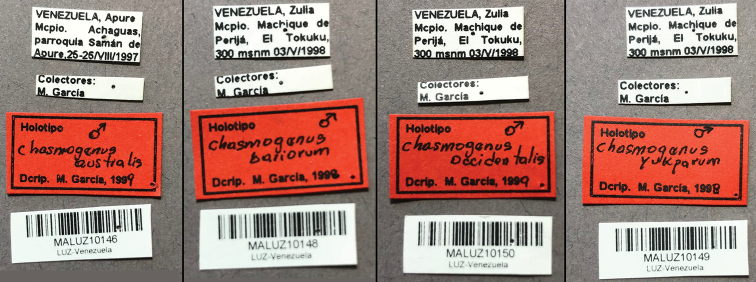
Holotype labels of the four Venezuelan species of *Chasmogenus* described by García (2000).

Figures 4D–F , 6 , 8B , 13A–D , 18 , 20C–F

Chasmogenus australis García, 2000: 52.

##### Type material examined.

**Holotype (Male)**: “Venezuela, Apure/ Mcipo. Achaguas, / parroquia Saman de/ Apure, 25–26/VIII/1997”, “Colectores:/ M. García”, “[Barcode]/ MALUZ10146/ LUZ-Venezuela”, “Holotipo [male symbol]/ Chasmogenus/ australis/ Dcrip. M. García, 1999” (MALUZ). The label data of the holotype (Fig. 6) differs slightly from that given in the original description. The locality is identical as listed in García (2000), but the date and collector data differ (given as 13 August 1998 and M. García & E. Gomez). The type is an undissected male, the aedeagus is visibly protruding from the abdomen. We also examined a permanent genitalia slide that is labeled as this species (Fig. 13A).

##### Additional material examined.

**Brazil: Roraima**: Circa 30 km southeast of Caracarai on BR-174, 1°35.091'N, 61°00.118'W, 80 m, 16.i.2018, leg. Short, Benetti, and Santana, marsh, BR18-0116-05A (37 exs., INPA, SEMC, including DNA Voucher SLE1774); Murupu River at BR-174 north of Boa Vista, 3°01.276'N, 60°46.565'W, 75 m, 13.i.2018, leg. A. E. Z. Short, muddy backwaters of stream, BR18-0113-01A (3 exs., SEMC); Vicinal 30, 00°43'50.2"N, 060°25'58"W, 77 m, 10.i.2018, leg. Short and Benetti, large roadside ditch with dense vegetation, BR18-0110-03A (24 exs., INPA, SEMC, including DNA voucher SLE1624); ca. 16 km west of Amajari on BR-203, 3°36.874'N, 61°33.470'W, 125 m, 13.i.2018, leg. Short, Benetti, and Santana, marsh, BR18-0113-04A (9 exs., SEMC); Circa 26 km south of Cantá, 2°22.547'N, 60°33.538'W, 93 m, 11.i.2018, leg. Short, marsh with lots of emergent vegetation, BR18-0111-02A (1 ex., SEMC); Circa 7 km south of Iracema on BR-174, 2°6.277'N, 61°4.922'W, 59 m, 16.i.2018, leg. Short, Benetti, and Santana, marsh and palm swamp, BR18-0116-01A (1 ex., SEMC); Jundia on BR-174, 00°12'20.3"S, 060°41'35.1"W, 57 m, 10.i.2018, leg. Short and Benetti, marsh with lots of emergent vegetation, BR18-0110-01A (1 ex.., SEMC); Sitio Bem Querer ca. 3 km west, 1°56.131'N, 61°01.737'W, 80 m, 16.i.2018, leg. Short, Benetti, and Santana, BR18-0116-04A (2 exs., SEMC); near Boa Vista, 2°44.558'N, 60°47.179'W, 105 m, 15.i.2018, leg. Short, Benetti, and Santana, UV light by drying marsh, BR18-0115-03A, (1 ex., SEMC); BR-174, c. 50 km NW Boa Vista, 3 18.348'N, 60 51.458'W, 100 m, 13.i.2018, leg. Short, marsh, BR18-0113-02A (1 ex., SEMC, DNA Voucher 1629). **French Guiana**: Yiyi, 5.419, -53.050 (1 ex., SEMC, DNA Voucher SLE1856). **Guyana: Region 9**: near Kusad Mountains, 2°52.204'N, 59°55.003'W, 124 m, 27.x.2013, leg. Short, Isaacs, and Salisbury, marshy area, GY13-1027-01A (2 exs., SEMC); Ziida Wao Creek near Kusad Mountains, 2°49.724'N, 59°48.546'W, 121 m, 25.x.2013, leg. Short, Isaacs, and Salisbury, stagnant vegetated creek, GY13-1025-02A (3 exs., CBDG, SEMC, including DNA Voucher SLE1615); **Venezuela: Barinas**: ca. 13 km southeast of Ciudad Bolivia, 8°19.394'N, 70°28.238'W, 173 m, 25.i.2012, leg. Short, Arias, and Gustafson, marsh, VZ12-0125-02A (6 exs., SEMC, including DNA voucher SLE1080). **Cojedes**: Rio Caiman Grande at San Brano, 9°39.246'N, 68°11.860'W, 137m, 20.i.2012, leg. Short, Arias, and Gustafson, river margin, VZ12-0120-03A (2 exs., SEMC). **Monagas**: South of Maturin, 9°16.398'N, 62°56.246'W, 22 m, 2.ii.2010, leg. Short, García, and Joly, morichal margin, VZ10-0202-02A (6 exs., SEMC); between Morichal Largo and Tembledor, 9°05.798'N, 62°43.618'W, 29 m, 2.ii.2010, leg. Short, García, and Joly, margins of vegetated pond, VZ10-0202-03A (11 exs., MIZA, SEMC; including DNA voucher SLE1621). **Zulia**: Sabana de Machango, 10.043017, -71.007133, 35 m, 29.i.2012, leg. Short, Arias and Gustafson, margin of artificial pond, VZ12-0129-03A (1 ex., SEMC, DNA Voucher SLE1082).

##### Differential diagnosis.

The small size (<3.5 mm) and very pale dorsal coloration (Fig. 4D–F) serves to separate *C. australis* from most other small-bodied regional congeners, as well as its distinctive aedeagus. It also almost exclusively occurs in open marsh habitats, whereas most all other congeners are typically found in other types of habitat.

##### Description.

***Size and color.*** Total body length 3.0–3.4 mm. Body form elongate oval with slightly curved lateral margins. Dorsum of head bicolored; frons dark brown, clypeus and labrum light tan-brown (Fig. 8B). Pronotum and elytra light tan-orange, light golden yellow marginally (Fig. 4D). Venter almost entirely light golden-brown, venter of head and lateral margins of metaventrite light brown (Fig. 4F). Tibiae orange-brown. ***Head.*** Ground punctation on head fine, labrum with slightly finer punctation. Clypeus with anteromedial emargination, which exposes a wide trapezoidal-shaped gap between clypeus and labrum (Fig. 8B). Mentum weakly to moderately depressed on anterior half with subtriangular anteromedial notch. Maxillary palps long, longer than width of head immediately posterior to eyes. ***Thorax.*** Ground punctation of pronotum moderate. Prosternum tectiform. Mesoventrite with weak elevation forming a thin posteromedial longitudinal carina. Metafemora densely pubescent with long golden setae in basal six-sevenths (Fig. 4F). ***Aedeagus.*** Aedeagus (Fig. 13A–D) with outer margins of median lobe strongly situate, such that it appears constricted in the middle; above constriction, margins tapering to form a weakly pointed apex, which slightly extends beyond the apex of the parameres. Sclerite of the median lobe not expanded. Gonopore situated less than half of one gonopore width below the apex of the median lobe. Parameres with outer margins weakly and evenly curved (Fig. 13A) to slightly sinuate near the apex (Fig. 14B–D), with outer apex bluntly rounded and inner apex appear as a right angle. Basal piece long, subequal to slightly shorter than the length of the parameres.

##### Distribution.

This species has a very broad range, occurring from northwestern Venezuela to the coast of French Guiana (Fig. 18). It was originally described from the central region of the Venezuelan Llanos. We have collected it across a very broad range in Venezuela, including the Maracaibo Basin, across the Llanos region, as well as in the Rupununi/Roraima savannah complex in northern Brazil and Guyana. We also report it from the coastal savannahs of French Guiana.

##### Biology.

Unlike most *Chasmogenus*, *C. australis* is almost exclusively associated with lentic or open marsh habitats (Fig. 20C–F). A few specimens have been taken along the muddy and vegetated margins of large, slow-flowing rivers (morichales).

##### Remarks.

Despite its broad range, sequenced populations of *C. australis* displayed virtually no genetic variation in COI, with less than 1% divergence between all sampled individuals spanning more than 2000 km from western Venezuela to French Guiana, south to the state of Roraima, Brazil. We also note the strong similarities in aedeagal morphology and habitat to *C. sapucay* Fernández, which was originally described from Argentina and Paraguay but whose range was recently extended into Brazil (Fernández 1986, Clarkson and Ferreira-Jr 2014). Although we suspect *C. australis* may be a junior subjective synonym of *C. sapucay*, we are refraining from making any nomenclatural changes until the two taxa can be compared in more detail.

#### Chasmogenus bariorum

Taxon classificationAnimaliaColeopteraHydrophilidae

García, 2000

636C8FB1-9559-580E-8265-20422D880B66

**Figure 7.**
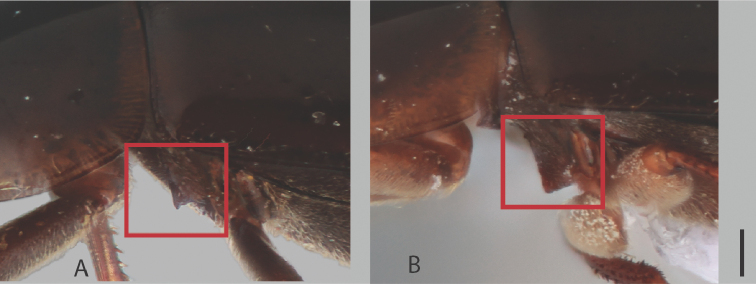
Lateral view of mesoventrite. **A***C. bariorum***B***C. cuspifer*. Scale bar: 0.10 mm.

**Figure 8.**
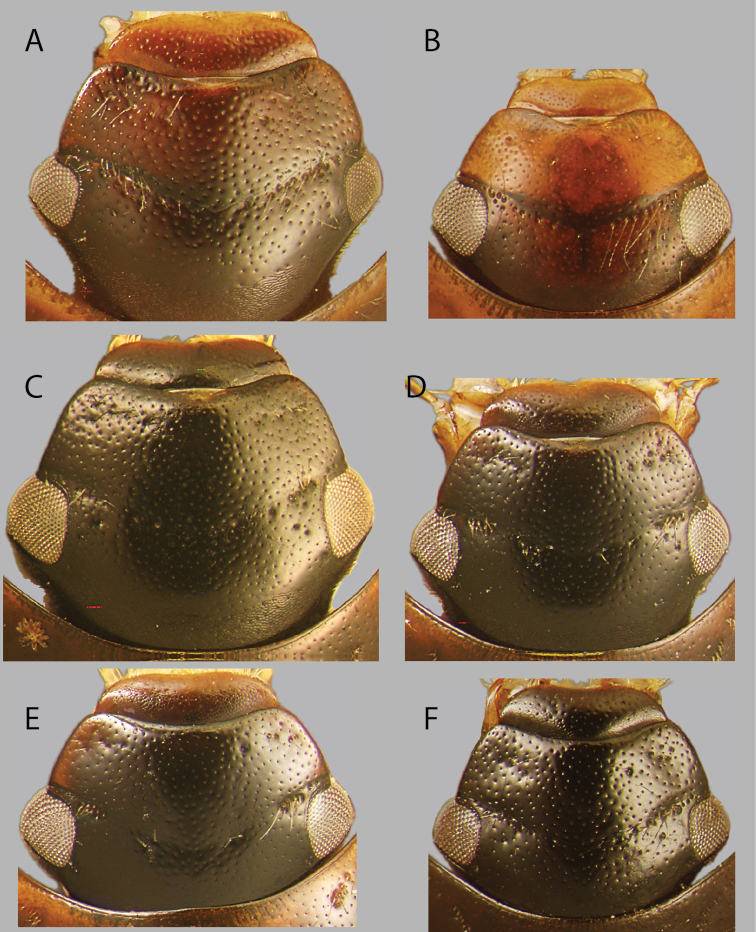
Dorsal view of heads of *Chasmogenus* spp. **A***C. castaneus***B***C. australis***C***C. bariorum***D***C. cuspifer***E***C. flavomarginatus***F***C. lineatus*.

Figures 3D–F , 6 , 7A , 8C , 11A–H , 17 , 19A–C , 19E, F

Chasmogenus bariorum García, 2000: 49.

##### Synonyms.

*Chasmogenus occidentalis* García, 2000, syn. nov. Type material examined: Holotype (male): “Venezuela, Zulia/Mcpio. Machique de/ Perija, El Tokuku,/300 msnm 03/V/1998”, “Colectores:/M. García”, “[Barcode]/ MALUZ10150/ LUZ-Venezuela”, “Holotipo [‘male symbol’/ Chasmogenus/ occidentalis/ Dcrip. M. García, 1999” (MALUZ). The date on the label of the holotype (Fig. 6) differs slightly from that given in the original description, which was listed as 2 May 1995 instead of 3 May 1998. The type is an undissected male, the aedeagus is visibly protruding from the abdomen. We also examined a permanent genitalia slide that had been presumed to be the holotype and is labeled as this species (Fig. 11B).

*Chasmogenus yukparum* García, 2000, syn. nov. Type material examined: Holotype (male): “Venezuela, Zulia/Mcpio. Machique de/ Perija, El Tokuku,/300 msnm 03/V/1998”, “Colectores:/M. García”, “[Barcode]/ MALUZ10149/ LUZ-Venezuela”, “Holotipo [‘male symbol’/ Chasmogenus/ yukparum/ Dcrip. M. García, 1998” (MALUZ). The date on the label of the holotype (Fig. 6) differs slightly from that given in the original description, which was listed as 5 May 1995 instead of 3 May 1998. The dorsal portion of the abdomen is missing and may be the result of dissection. We also examined a permanent genitalia slide which is labeled as this species and had been presumed to be the holotype (Fig. 11C). The description of this species was based on a single male so we assume the genitalia on the slide is the holotype.

##### Type material examined.

**Holotype (male)**: “Venezuela, Zulia/Mcpio. Machique de/ Perija, El Tokuku,/300 msnm 03/V/1998”, “Colectores:/M. García”, “[Barcode]/ MALUZ10148/ LUZ-Venezuela”, “Holotipo [‘male symbol’/ Chasmogenus/ bariorum/ Dcrip. M. García, 1998” (MALUZ). The date on the label of the holotype (Fig. 6) differs slightly from that given in the original description, which was listed as 2 May 1995 instead of 3 May 1998. The type is an undissected male, the aedeagus is visibly protruding from the abdomen. We also examined a permanent genitalia slide that had been presumed to be the holotype and is labeled as this species (Fig. 11A).

##### Additional material examined (198).

**Venezuela: Aragua**: Henri Pittier National Park, Río La Trilla, 10.37319°N, 67.74250°W, 295 m, 4.i.2009, Short, Miller, Camacho, & Garíca [sic], pools, VZ09-0104-01A (25 exs., MIZA, SEMC, including DNA Vouchers SLE078 and SLE531); Henri Pittier National Park, Río Cumboto, 10.39376N, 67.79597W, 130m, 4.i.2009, leg. Short, García, & Miller, riverside pools, VZ09-0104-02B (7 exs., SEMC); Henri Pittier National Park, 10°21.017'N, 67°40.883'W, 5.i.2009, leg. Miller, Camacho, and García, small stream, VZ09-0105-01A (1 ex., SEMC). **Guárico**: Río San Antonio, north of Dos Caminos, 9°46.320'N, 67°21.177'W, 280 m, 8.i.2009, leg. Short, Miller and García, river margins, VZ09-0108-02A (7 exs., SEMC); same as previous except: leg. Miller & Short, side stream, VZ09-0108-02B (8 exs., SEMC, including DNA Voucher SLE1613); same as previous except: leg. K. B. Miller, micro habitats, VZ09-0108-02C (2 exs., SEMC); Stream at road crossing, north of Palenque, 9°6.794'N, 66°59.595'W, 152m, 8.i.2009, leg. Short, García, Miller, Camacho and Joly, stream, VZ09-0108-03X (3 exs, SEMC); Río Guárico, N. San Juan, 9.95788N, 67.37773W, 435 m, 8.i.2009, leg. K. Miller & L. Joly, along river, VZ09-0108-01X (5 exs., SEMC); ~15 km south of San Juan, 9°46.321'N, 67°21.201'W, 255 m, 3.i.2006, leg. A. E. Z. Short, stream margin and rock pools, AS-06-005 (1 ex., SEMC); ~20 km north of Dos Caminos, 9°44.034'N, 67°19.003'W, 225 m, leg. A. E. Z. Short, gravelly margin of river, AS-06-020 (24 exs., SEMC). **Falcón**: Rio Ricoa near Dos Bocas, 11°17.424'N, 69°26.061'W, 170 m, 9.vii.2009, leg. Short, Sites, Gustafson, García, Camacho, and Inciarte, along river margins, VZ09-0709-02A/L-1063 (4 exs., SEMC). **Lara**: Río Salado west of Arenales, 10°9.260'N, 69°57.458'W, 490 m, 22.i.2009, leg. Short, García, and Camacho, gravel stream, VZ09-0122-01X (5 exs., SEMC). **Trujillo**: Rio Jirajara near Sabana Grande, 9°42.307'N, 70°32.570'W, 199 m, 29.i.2012, leg. Short, Arias, and Gustafson, muddy pool in floodplain, VZ12-0129-02A (4 exs., SEMC). **Zulia**: Perija National Park Tukuko, Río Manantial, 9°50.490'N, 72°49.310'W, 270 m, 29.i.2009, leg. Short, García, and Camacho, gravel margin, VZ09-0129-01A (13 exs., SEMC, including DNA Voucher SLE534); same data as previous except: 22.ix.2007, leg. A. E. Z. Short, rock pools/margins, AS-07-020b (27 exs., SEMC); same data as previous except: 16.vii.2008, leg A. Short, margins and pools, AS-08-027 (14 exs., SEMC); Perija National Park, Rio Tukuko, 09°50.515'N, 72°48.334'W, 15.vii.2008, leg. A. E. Z. Short, upstream of Tukuko, AS-08-029 (1 ex., SEMC); Perija National Park, Toromo, 10°03.058'N, 72°42.974'W, 435 m, 31.xii.2005, leg. A. E. Z. Short, small stream and seep, AS-06-001 (5 exs., SEMC); same data as previous except: 28.i.2009, leg. A. Short, detrital pool, VZ09-0128-01A (3 exs., SEMC); c. 15 km southwest of El Dibujo, 10.79307N, 72.32331W, 155 m, 30.xii.2008, leg. Short, García, and Camacho, muddy puddle, VZ08-1230-03B (1 ex, SEMC); same data as previous except: in stream, VZ08-1230-03X (2 exs., SEMC); Marshy pond, 10.85498N, 72.30837W, 81 m, 30.xii.2008, leg. Short, García, and Camacho, pond margin, VZ08-1230-02X (1 ex., SEMC); 15 km west of Machiques, 10°02.962'N, 72°42.615'W, 432 m, 31.xii.2005, leg. A. E. Z. Short, isolated rock pool, AS-06-002 (1 ex., SEMC); Quebrada Riencito, 10.86041N, 72.32210W, 95 m, 30.xii.2008, leg. A. Short and M. García, along margin, VZ08-1230-01B (34 exs., MIZA, SEMC).

##### Differential diagnosis.

This species may be easily distinguished from others in the region by mesoventrite raised into an acute tooth (Fig. 7A), shared only with the sympatric *C. cuspifer*. *Chasmogenus bariorum* can be separated from *C. cuspifer* by its larger size (≥ 3.5 mm) and narrower apex of the median lobe (11A–H).

##### Description.

***Size and color.*** Total body length 3.5–3.7 mm. Body form elongate oval with slightly subparallel lateral margins. Dorsum of head very dark brown to black, anterior margin of labrum slightly paler in color (Fig. 8C). Pronotum dark brown, distinctly paler at anterior and lateral margins; elytra dark brown, slightly paler at posterior margins (Fig. 3D). Prosternum and abdominal ventrites dark brown; meso- and metaventrites dark red-brown, trochanters and glabrous portion of femora red-orange (Fig. 3F). ***Head.*** Ground punctation on head fine. Clypeus with anteromedial emargination, which exposes a trapezoidal-shaped gap between clypeus and labrum (Fig. 8C). Mentum weakly depressed in anterior half with shallowly rounded anteromedial notch. Maxillary palps long, longer than width of head immediately posterior to eyes. ***Thorax.*** Ground punctation of pronotum fine. Prosternum moderately tectiform. Mesoventrite with median longitudinal carina, which is elevated into an acute tooth medially (Fig. 7A). Metafemora densely pubescent with long golden setae in basal four-fifths (Fig. 3F). ***Aedeagus.*** Aedeagus (Fig. 11A–H) with outer margins of median lobe straight and parallel sided, with apex in the form of an acutely pointed triangle, which distinctly extends beyond the apex of the parameres. Sclerite of the median lobe not expanded. Gonopore situated less than half of one gonopore width below the apex of the median lobe. Parameres symmetrical, with outer margins slightly curved along entire length, appearing weakly convex; apex bifid, with outer and inner lobes subequal in height but with inner lobe usually narrower. Basal piece long, subequal to the length of the parameres.

##### Distribution.

Venezuela (Aragua, Falcón, Guárico, Lara, Trujillo, Zulia) (Fig. 17).

##### Biology.

Nearly all specimens are associated with the margins or side pools of streams and small rivers in the foothills of various Andean regions of Venezuela up to elevations of ca. 500 m. (Fig. 19A–C, 19E, F)

##### Remarks.

García (2000) described three species from the Rio Manantial, a small forested stream near Tokuko in the Serranía de Perijá in northwestern Venezuela. We compared the holotypes of *C. bariorum*, *C. occidentalis*, and *C. yukparum* and determined they are conspecific. Because all three were described in the same publication, we here select *C. bariorum* as the valid name based on the principal of first reviser.

The shape of the mesoventral tooth was used as a primary character to separate these three species but after examining specimens from a variety of localities we found this character to be variable. The presence of only a single species despite the variability of this feature is also supported by genetic data (Fig. 1). In his species descriptions, García (2000) further differentiates *C. yukparum* from *C. bariorum* and *C. occidentalis* by indicating that the former has asymmetrical mandibles, while the latter two exhibit symmetrical mandibles; however, in the identification key this character is reversed, with the mandibles of *C. occidentalis* being described as asymmetrical while those of *C. yukparum* are symmetrical. Regardless, we examined the mandibles in the three holotypes and found no substantial difference in mandibular symmetry; all three types have bifid mandibles with minor variation in the size of the teeth, as with many of the other specimens we examined.

There appears to be some confusion with regard to the genitalia slides associated with the holotypes. In García (2000), the caption of fig. 3 indicates all illustrated genitalia are of the holotypes. However, this is not possible as some of the labeled holotypes are undissected males. Because *C. yukparum* was described from a single male and its abdomen is partially missing, we presume the genitalia slide associated with the holotype is in fact the holotype. However, we are uncertain of which exact specimens should be associated with the “type” genitalia slides of *C. bariorum* and *C. occidentalis*. Regardless, this uncertain association does not impact any of our conclusions regarding their synonymy: it is clear that all three genitalia slides represent a single species (*C. bariorum*) and that all three male holotype specimens represent the same conspecific species as those on the slides.

#### Chasmogenus berbicensis sp. nov.

Taxon classificationAnimaliaColeopteraHydrophilidae

EAFAA4B4-9F69-58FC-B4B6-DA60431550FF

http://zoobank.org/58124959-D58E-4CF6-9815-D6ED103DDF7B

**Figure 9.**
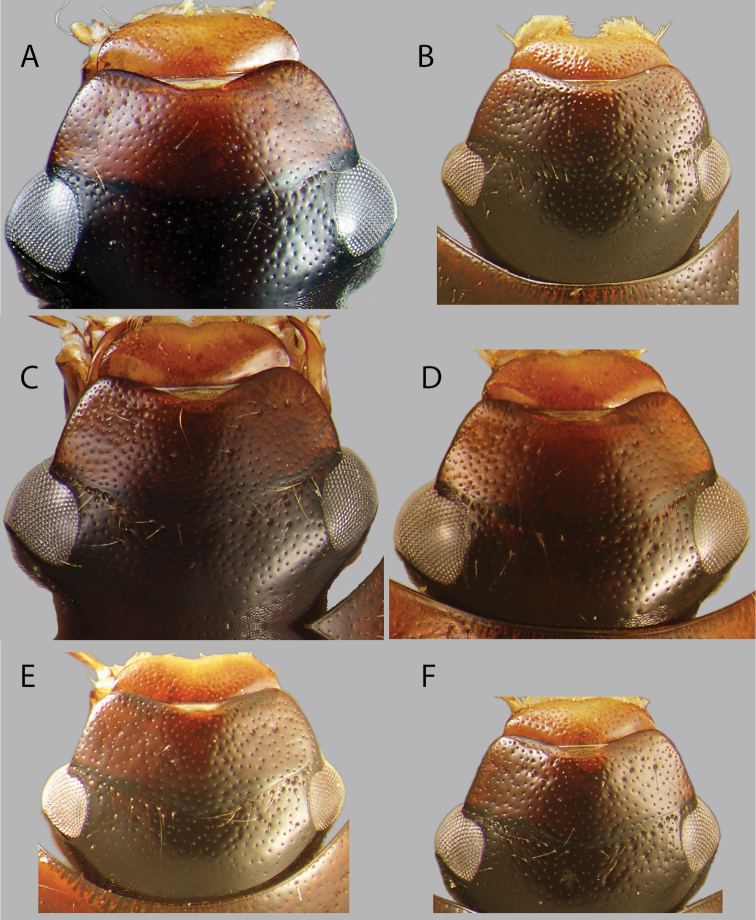
Dorsal view of heads of *Chasmogenus* spp. **A***C. amplius***B***C. acuminatus***C***C. berbicensis***D***C. clavijoi***E***C. undulatus***F***C. gato*.

Figures 9C , 14D , 17 , 22A

Chasmogenus sp. B Short, Salisbury, & La Cruz 2018: 193.

##### Type material.

**Holotype (male)**: “Guyana: Region 6/ 4°08.809'N, 58°14.232'W, 108/ Upper Berbice, Basecamp 1/ margin of berbice river/ leg. Short, Salisbury, La Cruz/ 22.ix.2014; GY14-0922-02A”/ “[barcode] SEMC1357824/ KUNHM-ENT”, “HOLOTYPE/ CHASMOGENUS/ berbicensis sp. n./ des. Smith & Short” (CBDG). **Paratypes (6): Guyana: Region 6**: same data as holotype (3 exs., SEMC including DNA Voucher SLE1864); Upper Berbice Basecamp 1m 4°09.289'N, 58°10.717'W, 96 m, 24.ix.2014, leg. Short, Salisbury, and La Cruz, margins of basecamp creek, GY14-0924-01A (2 exs., SEMC); same data as previous except, 21.ix.2014, leg. Short Salisbury and La Cruz, muddy detrital pools in drying creek bed near camp, GY14-0921-02A (1 ex., SEMC).

##### Differential diagnosis.

See differential diagnosis for *C. amplius*.

##### Description.

***Size and color.*** Total body length 4.5–4.9 mm. Body form elongate oval with slightly curved lateral margins. Dorsum of head multi-colored, frons dark brown, clypeus dark orange-brown, labrum pale yellow-orange (Fig. 9C). Pronotum red-orange, elytra pale orange-brown with serial dark brown spots. ***Head.*** Ground punctation on head fine. Clypeus with anteroposterior emargination that exposes a wide trapezoidal shaped gap between clypeus and labrum (Fig. 9C). Mentum moderately depressed in anterior half with subtriangular anteromedial notch. Maxillary palps long, longer than width of head immediately posterior to eyes. ***Thorax.*** Ground punctation on pronotum fine. Prosternum mildly tectiform. Mesoventrite with weak elevation forming a posteromedial longitudinal carina. Metafemora densely pubescent in basal nine-tenths. ***Aedeagus.*** Aedeagus (Fig. 14D) with median lobe widest at base, and very slightly tapering until the apical quarter, then narrowing abruptly to form an acute triangular apex which is even with or appears very slightly extended beyond the apex of the parameres. Sclerite of the median lobe not expanded. Gonopore situated less than half of one gonopore width below the apex of the median lobe. Parameres symmetrical, with outer margins straight, with apex slightly inwardly curved and bluntly rounded. Basal piece long, ca. four-fifths the length of the parameres.

##### Etymology.

The species is named after the Berbice River in Guyana from where was collected. To be treated as a noun in apposition.

##### Distribution.

Known only from the type locality along the upper Berbice River in Guyana (Fig. 17).

##### Biology.

This species was collected in detrital leaf packs along the margin of the upper Berbice River (Fig. 22A).

#### Chasmogenus brownsbergensis sp. nov.

Taxon classificationAnimaliaColeopteraHydrophilidae

267ACFB7-D38C-5390-96C8-3C6CE6189456

http://zoobank.org/91D796FA-D130-4970-BF69-524AEB0050C7

**Figure 10.**
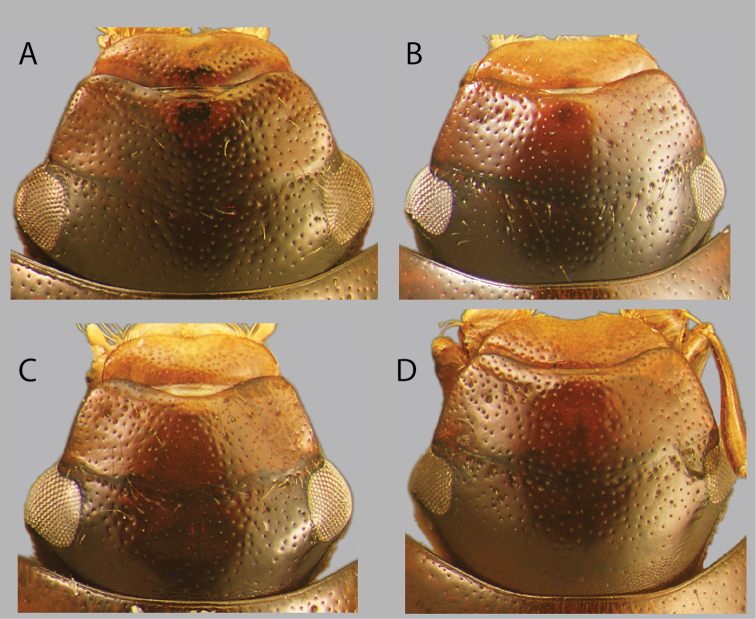
Dorsal view of heads of *Chasmogenus* spp. from Suriname and Guyana **A***C. brownsbergensis***B***C. ligulatus***C***C. guianensis***D***C. pandus*.

Figures 10A , 14E , 17 , 21A

##### Type material.

**Holotype (male)**: “Suriname: Brokopondo District/ 04°56.871'N, 55°10.911'W, 462 m/ Brownsberg Nature Park, forested/ stream with lots of detritus; leg./ Short, Maier, McIntosh; 4.viii.2012/ SR12-0804-01A”, “[barcode]/SEMC1114121/ KUNHM-ENT” “HOLOTYPE/ CHASMOGENUS/ brownsbergensis sp. n./ des. Smith & Short” (NZCS). **Paratypes (46): Suriname : Brokopondo District**: same data as holotype except: 4.viii.2012, pools in road, SR12-0804-03A (20 exs., SEMC, including DNA Voucher SLE1828); Brownsberg Nature Park, trail between Park HQ and Mazaroni Val, 4°56.934'N, 55°10.825'W, 467 m, 20.iii.2017, leg. Short et al., pools in dirt road, SR17-0320-02A (18 exs., SEMC, NZCS); same data as previous except: 22.iii.2017, SR17-0322-01A (8 exs., SEMC, including DNA Voucher SLE1861).

##### Differential diagnosis.

Among smaller species with a broadly rounded clypeal emargination, this species is similar in size, morphology, and general form of the aedeagus as *C. guianensis*, but can be distinguished by the coloration of the head which has mesal dark patches on the clypeus and labrum (Fig. 10A) in contrast to the typical bicolored or uniform coloration of the head. In addition, the apex of the median lobe of the aedeagus is ca. the same level as the apex of the parameres. In *C. guianensis*, the apex of the median lobe is shorter than the apex of the parameres.

##### Description.

***Size and color.*** Total body length 3.7–3.8 mm. Body form elongate oval with slightly curved lateral margins. Dorsum of head bicolored, frons very dark brown, labrum and clypeus dark orange-brown (Fig. 10A). Darker patches on mesal portions of clypeus and labrum (Fig. 10A). Elytra uniformly dark brown. Venter dark orange-brown centrally, dark brown marginally. ***Head.*** Ground punctation on head moderately coarse. Clypeus with anteromedial emargination, which exposes a wide smoothly rounded gap between labrum and clypeus (Fig. 10A). Mentum strongly depressed in anterior half with subtriangular anteromedial notch. Maxillary palps long, longer than width of head immediately posterior to eyes. ***Thorax.*** Ground punctation on pronotum moderately coarse. Prosternum even, not tectiform. Mesoventrite with very weak elevation forming a thin posteromedial longitudinal carina. Metafemora densely pubescent in basal nine-tenths. ***Aedeagus.*** Aedeagus (Fig. 14E) with median lobe widest at base and very slightly tapering until the apical fifth, then narrowing to form an acute triangular apex which is even with the apex of the parameres. Sclerite of the median lobe not expanded. Gonopore situated ca. half of one gonopore width below the apex of the median lobe. Parameres symmetrical, with outer margins straight, with apex inwardly curved, tapered, and bluntly rounded. Basal piece of medium length, ca. two-thirds the length of the parameres.

##### Etymology.

The species is named after Brownsberg Nature Park, the only locality where it is currently known. To be treated as a noun in apposition.

##### Distribution.

This species is only known from Brownsberg Nature Park in Suriname (Fig. 17).

##### Biology.

Long series of this species were collected from pools in dirt roads in Brownsberg Nature Park. The pools are large, and often contain detritus as they are through forested areas (Fig. 21A). One specimen was collected in a forested stream with lots of detritus.

**Figure 11.**
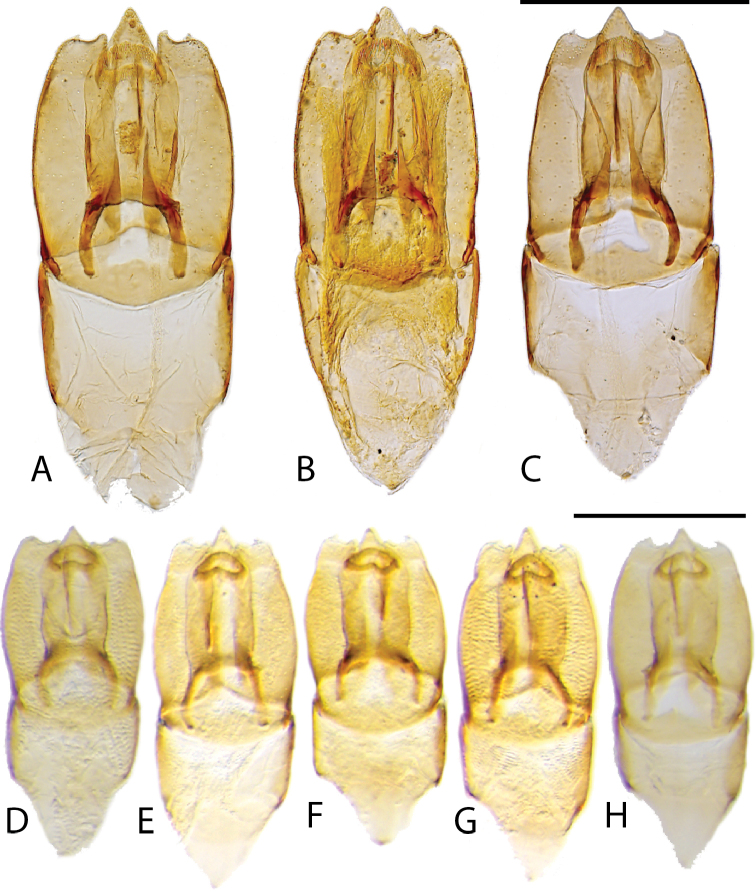
Aedeagi (dorsal view) **A***C. bariorum* holotype (Venezuela: Zulia) **B***C. occidentalis* holotype **C***C. yukparum* holotype **D–H***C. bariorum***D** specimen from Zulia **E** specimen from Aragua **F** specimen from Falcón **G** specimen from Guárico **H** specimen from Trujillo. Scale bars: 0.25 mm.

#### Chasmogenus castaneus sp. nov.

Taxon classificationAnimaliaColeopteraHydrophilidae

68AF2A45-BDA6-5230-9A01-1844F1D588BD

http://zoobank.org/8664C494-2BBD-455F-AB19-72431B581C13

**Figure 12.**
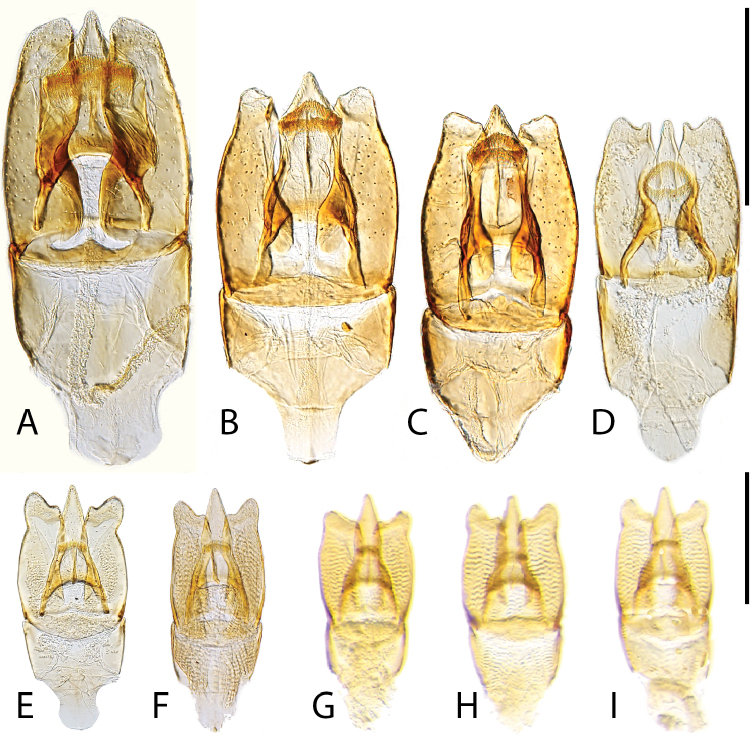
Aedeagi (dorsal view) of *Chasmogenus* spp. **A***C. castaneus***B***C. flavomarginatus***C***C. flavomarginatus***D***C. cuspifer***E–I***C. lineatus*. Scale bar: 0.25 mm.

Figures 3G–I , 8A , 12A , 17 , 19A, B

##### Type material.

**Holotype (male)**: “Venezuela: Zulia State/ 09°50.490'N, 72°49.310'W; 270m/ Perija National Park, Tukuko,/ Rio Manantial: margins and pools/ 16.vii.2008/ leg. A. Short; AS-08-027”, “[barcode]/ SEMC0929744/ KUNHM-ENT”, “HOLOTYPE/ CHASMOGENUS/ castaneus sp. n./ des. Smith & Short” (MIZA). **Paratypes (3): Venezuela: Zulia**: Same data as holotype (3 exs., SEMC, including DNA voucher SLE1779).

##### Differential diagnosis.

From other Andean species with a triangular clypeal emargination, *C. castaneus* can be differentiated by its larger size (> 4.5 mm) and its distinct chestnut dorsal coloration (Fig. 3G–I). The overall body form with parallel sides is similar to *C. lineatus*, but that species is much smaller and entirely dark brown to black in coloration. In addition, the apex of the parameres are not sinuated.

##### Description.

***Size and color.*** Total body length 4.0 mm. Body form elongate oval with slightly subparallel lateral margins. Dorsum of head bicolored, frons dark brown to mottled orange-brown (Fig. 8A), anterior margin of labrum and clypeus slightly paler in color. Pronotum mottled dark orange and dark brown, paler on lateral anterior margin (Fig. 3G). Elytra orange-brown, lateral margins very dark brown (Fig. 3G). Venter of head dark brown to dark red-brown. Maxillary palps and tarsi exhibit proximodistal gradation from light brown to light tannish yellow. Venter dark red-brown (Fig. 3I). ***Head.*** Ground punctation on head fine. Clypeus with anteromedial emargination which exposes a wide rounded to subtriangular gap between labrum and clypeus (Fig. 8A). Mentum strongly depressed in anterior half with rounded anteromedial notch. Maxillary palps long, longer than width of head immediately posterior to eyes. ***Thorax.*** Ground punctation on pronotum moderately coarse. Prosternum tectiform. Mesoventrite with broad elevation posteromedially, forming a posteromedial longitudinal carina. Metafemora densely pubescent in basal six-sevenths (Fig. 3I). ***Aedeagus.*** Aedeagus (Fig. 12A) with median lobe very wide at base, wider than the width of one paramere, tapering abruptly in apical quarter to form a narrow triangular projection which is even with to slightly extending beyond the apex of the parameres. Sclerite of the median lobe not expanded. Gonopore situated ca. half of one gonopore width below the apex of the median lobe. Parameres symmetrical, with outer margins slightly curved along entire length, appearing weakly convex; apex weakly angled inward. Basal piece long, slightly shorter than the length of the parameres.

##### Etymology.

The species name is derived from the Latin *castaneus*, meaning “of the color of chestnuts”, a reference to the distinctive reddish brown dorsal coloration of this species.

##### Distribution.

Only known from the type locality in northwestern Venezuela (Fig. 17).

##### Biology.

All specimens of *C. castaneus* were collected in marginal pools by a stream (Fig. 19A, B).

**Figure 13.**
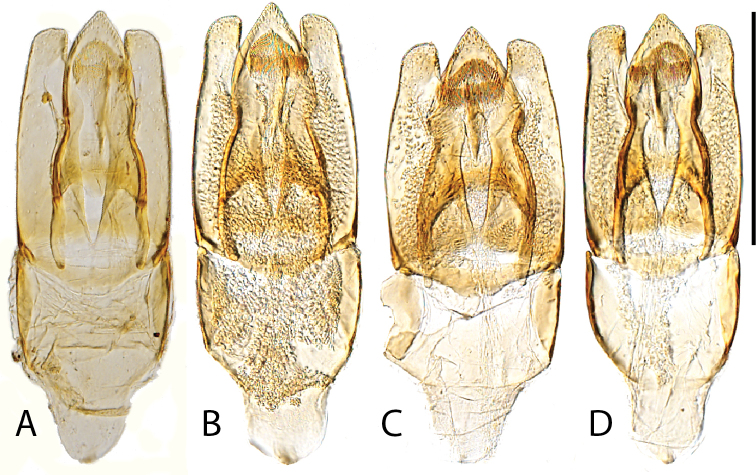
Aedeagi (dorsal view) of *C. australis***A** holotype (Venezuela: Apure) **B** specimen from Guyana (Region 9) **C** specimen from Venezuela (Monagas) **D** specimen from Brazil (Roraima). Scale bar: 0.25 mm.

#### Chasmogenus clavijoi sp. nov.

Taxon classificationAnimaliaColeopteraHydrophilidae

8953983E-25C9-5B6B-9AF3-2CE9DC81098E

http://zoobank.org/02C1D58C-E5EC-493D-A4E4-182972AD729B

**Figure 14.**
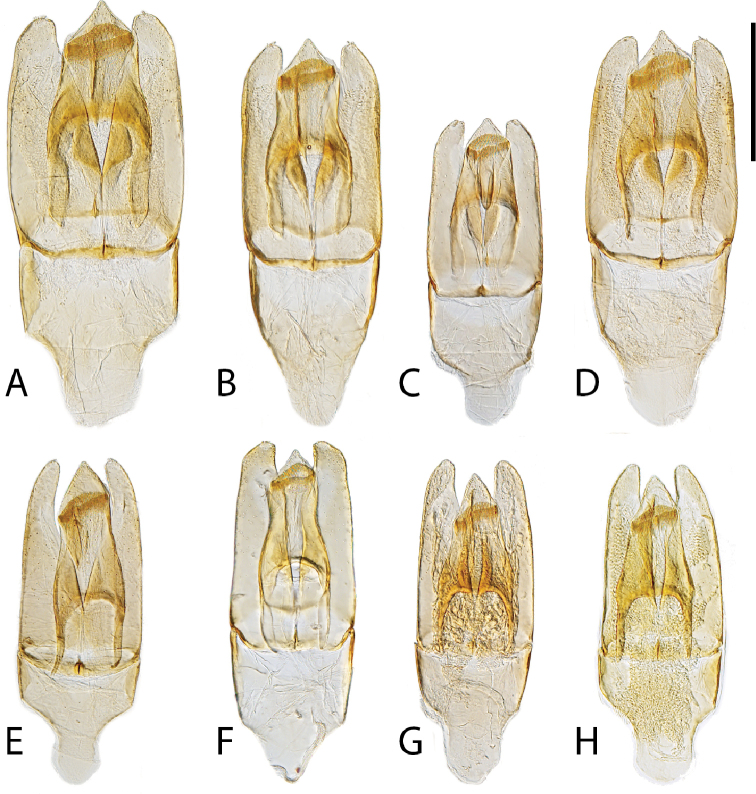
Aedeagi of (dorsal view) of *Chasmogenus* spp. **A***C. amplius***B***C. clavijoi***C***C. gato***D***C. berbicensis***E***C. brownsbergensis***F***C. schmits***G, H***C. guianensis***G** specimen from Suriname **H** specimen from Guyana. Scale bar: 0.25 mm.

Figures 9D , 14B , 17 , 20B

##### Material examined.

**Holotype (male)**: “Venezuela: Guárico Stat**e**/ 8°8.296'N, 66°24.459'W/ San Nicolasito Field Station/ 10.i.2009; leg. Short & Miller/ VZ09-0110-02X; morichal”/ “[barcode]/ SEMC0855289/ KUNHM-ENT”, “HOLOTYPE/ CHASMOGENUS/ clavijoi sp. n./ des. Smith & Short” (MIZA). **Paratypes (5)**: same data as holotype (5 exs. SEMC, including DNA voucher SLE1198).

##### Differential diagnosis.

Though *C. clavijoi* shares a smaller body size and similar morphology as *C. gato*, it may be differentiated by the paler dorsal coloration.

##### Description.

***Size and color.*** Total body length 3.8–4.5 mm. Body form elongate oval with slightly curved lateral margins. Dorsum of head bicolored, frons dark brown, clypeus and labrum orange-brown (Fig. 9D). Pronotum dark orange brown, slightly paler marginally. Elytra yellow-orange with serial dark brown spots. ***Head.*** Ground punctation on head fine. Clypeus with anteroposterior emargination which exposes a broadly rounded gap between clypeus and labrum (Fig. 9D). Mentum moderately depressed in anterior half with subtriangular anteromedial notch. Maxillary palps long, longer than width of head immediately posterior to eyes. ***Thorax.*** Ground punctation on pronotum fine. Prosternum slightly tectiform. Mesoventrite with weak elevation forming a posteromedial longitudinal carina. Metafemora densely pubescent in basal nine-tenths. ***Aedeagus.*** Aedeagus (Fig. 14B) with median lobe widest at base, appearing weakly constricted medially, slightly tapering until the apical quarter, then narrowing abruptly to form an acute triangular apex which is even with the apex of the parameres. Sclerite of the median lobe not expanded. Gonopore situated less than half of one gonopore width below the apex of the median lobe. Parameres symmetrical, with outer margins straight, with apex slightly inwardly curved and bluntly rounded. Basal piece long, ca. four-fifths the length of the parameres.

##### Etymology.

Named in honor of Venezuelan entomologist José (Pepe) Clavijo, retired director of MIZA, for all his contributions to Neotropical entomology. To be treated as a noun in apposition.

##### Distribution.

Known only from the San Nicolasito Research Station in the Llanos of Venezuela (Fig. 17).

##### Biology.

This species was collected along the margins of a morichal, a riparian habitat of slow-moving water through a savannah (Fig. 20B).

**Figure 15.**
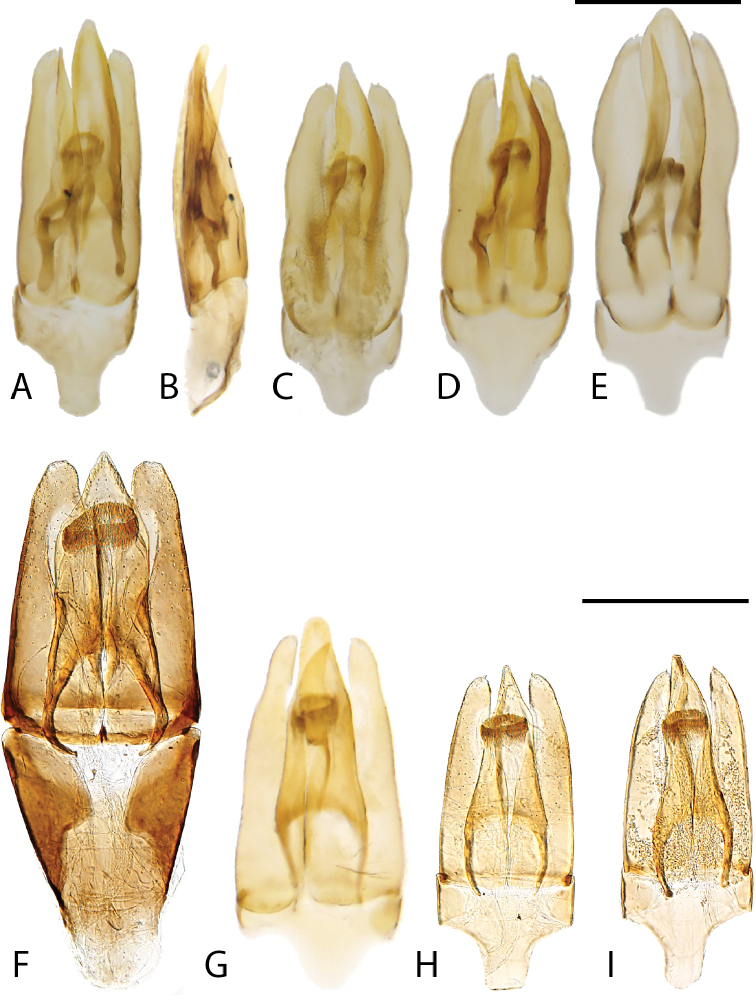
Aedeagi of *Chasmogenus* spp. **A–D***C. pandus***A** dorsal view (Suriname) **B** side view (Suriname) **C** specimen from French Guiana **D** specimen from Brazil **E***C. ligulatus***F***C. sinnamarensis***G***C. undulatus***H, I***C. acuminatus***H** specimen from Suriname **I** specimen from Guyana. Scale bars: 0.25 mm.

#### Chasmogenus cuspifer sp. nov.

Taxon classificationAnimaliaColeopteraHydrophilidae

FF4A3DD3-3E52-579A-902C-FE2273DE07F6

http://zoobank.org/7CB1B53B-4F0F-47A3-8DEA-527F2B6054BA

Figures 7B , 8D , 12D , 17 , 19A–C

##### Type material.

**Holotype (male)**: “Venezuela: Zulia State/ 9°50.490'N, 72°49.310'W, 270 m/ Perija N.P. Tukuko: Río Manantial/ 29.i.2009; Short, García, Camacho/VZ09-0129-01A: gravel margin”/ “[barcode]/SEMC0857741/ KUNHM-ENT”, “HOLOTYPE/ CHASMOGENUS/ cuspifer sp. n./ des. Smith & Short” (MIZA). **Paratypes (19): Venezuela: Aragua State**: Henri Pittier National Park, Rio La Trilla, 10.37319N, 67.74250W, 295 m, 4.i.2009, leg. Short, Miller, Camacho and García, pools, VZ09-0104-01A (1 ex., SEMC, DNA Voucher SLE532). **Zulia State**: Perija National Park, Tukuko: Rio Manantial, 9°50.490'N, 72°49.310'W, 270 m, 29.i.2009, leg. Short, García and Camacho, gravel margin, VZ09-0129-01A (11 exs., MIZA, SEMC, including DNA Voucher SLE533); same data as previous except: 16.vii.2008, leg. A. Short, margins and pools, AS-08-027 (2 exs., SEMC); same data as previous except: 22.ix.2007, rock pools/margin, leg. A. E. Z. Short, AS-07-020b (3 exs., SEMC); Perija National Park Toromo, 10°03.058'N, 72°42.974'W, 435 m, 31.xii.2005, leg. A. E. Z. Short, small stream and seep, AS-06-001 (1 ex., SEMC), same data as previous except: 28.i.2009, leg. A. Short, detrital pool, VZ09-0128-01A (1 ex., SEMC).

##### Differential diagnosis.

See differential diagnosis for *Chasmogenus bariorum*.

##### Description.

***Size and color.*** Total body length 3.0–3.2 mm. Body form elongate oval with slightly curved lateral margins. Dorsum of head very dark brown to black, anterior margin of labrum slightly paler in color (Fig. 8D). Pronotum dark brown, distinctly paler at lateral margins; elytra dark brown. Venter dark red-brown mesally, dark brown marginally. ***Head.*** Ground punctation on head fine. Clypeus with anteromedial emargination, which exposes a small angulate gap between clypeus and labrum (Fig. 8D). Mentum very weakly depressed in anterior half with widely rounded anteromedial notch followed by rounded elevated ridge just posterior of the notch. Maxillary palps long, longer than width of head immediately posterior to eyes. ***Thorax.*** Ground punctation of pronotum fine. Prosternum tectiform. Mesoventrite with strong elevation forming an acute tooth posteromedially (Fig. 7B). Metafemora densely pubescent in basal four-fifths. ***Aedeagus.*** Aedeagus (Fig. 12D) with median lobe subtriangular in shape, widest at base and gradually tapering along entire length; apex acute, which extends to the same level as the inner cusp of the parameres. Sclerite of the median lobe not expanded. Gonopore situated slightly less than one gonopore width below the apex of the median lobe. Parameres symmetrical, with outer margins slightly curved along entire length, appearing weakly convex; apex bifid, with outer lobe slightly more elevated than inner lobe; inner lobe much narrower than outer lobe. Basal piece very long, distinctly longer than the length of the parameres.

##### Etymology.

The species name is derived from the Latin *cuspis*, meaning “pointed”, after the toothed mesoventral carina.

##### Distribution.

This species is known from stream habitats in the Perijá and the Coastal Mountains of Venezuela (Fig. 17).

##### Biology.

All specimens were collected in the margins of forested stream habitats, typically with gravel margins (Fig. 19C).

#### Chasmogenus flavomarginatus sp. nov.

Taxon classificationAnimaliaColeopteraHydrophilidae

4C7A2BA0-1A8E-506B-B26F-CFC11D550A3F

http://zoobank.org/F6737BA7-61CB-453B-BF28-B7FA1830F4F0

Figures 3A–C , 8E , 12B, C , 17 , 19D

##### Type material.

**Holotype (male)**: “Venezuela: Barinas State/ 8°48.424'N, 70°31.139'W, 992m/ ca. 13km NW Baranitas, 24.i.2012/ leg. Short, Arias, & Gustafson/ Small stream pool: VZ12-0124-02B”, “[barcode]/ SEMC1030004/ KUNHM-ENT”, “HOLOTYPE/ CHASMOGENUS/ flavomarginatus sp. n./ des. Smith & Short” (MIZA). **Paratypes (88): Venezuela: Barinas**: Same data as holotype (85 exs., MIZA, SEMC, including DNA voucher SLE1235); same data as holotype except: seepage by road, VZ12-0124-02A (2 exs., SEMC, including DNA voucher SLE1083). **Táchira**: near El Tama, 26.i.2012, leg. Short, VZ12-0126-04A (1 ex., SEMC, DNA voucher SLE1084).

##### Differential diagnosis.

The lack of a clypeal emargination (Fig. 8E) easily separates *C. flavomarginatus* from most other congeners. It shares this characteristic with *C. lineatus* but can be separated by the overall paler dorsal coloration and the distinct yellow margins (Fig. 3A, B) of the pronotum (dorsum entirely dark brown to black in *C. lineatus*).

##### Description.

***Size and color.*** Total body length 3.2–3.5 mm. Body form elongate oval with slightly subparallel lateral margins. Dorsum of head very dark brown to black with slightly paler preocular patches (Fig. 8E). Anterior margin of labrum and lateral margins of clypeus sometimes slightly paler. Pronotum dark brown to black centrally, distinctly bright yellow at anterior and lateral margins (Fig. 3A–B). Elytra dark red-brown to dark brown, slightly paler or bright yellow marginally (Fig. 3A). Venter dark red brown, abdominal ventrites dark brown. ***Head.*** Ground punctation on head fine. Clypeus and labrum contiguous (Fig. 8E). Mentum weakly depressed in anterior half with shallowly rounded anteromedial notch. Maxillary palps short, just slightly shorter than width of head immediately posterior to eyes. ***Thorax.*** Ground punctation on pronotum fine. Prosternum moderately tectiform. Mesoventrite with elevation forming a posteromedial longitudinal carina with convex distolateral margins. Metafemora densely pubescent in basal four-fifths (Fig. 3C). ***Aedeagus.*** Aedeagus (Fig. 12B, C) with outer margins of median lobe straight and parallel sided, with apex in the form of an acutely pointed triangle, which slightly extends beyond the apex of the parameres. Sclerite of the median lobe not expanded. Gonopore situated less than half of one gonopore width below the apex of the median lobe. Parameres symmetrical, with outer margins slightly curved along basal four fifths, appearing weakly convex, then curved slightly outward at apex; apex very weakly bifid, with outer lobe more elevated than inner lobe; inner lobe minute, almost appearing absent. Basal piece long, ca. four-fifths the length of the parameres.

##### Variation.

In material examined, there was some variation in dorsal coloration, ranging from dark brown to black. There is also variation in the coloration of the dorsum of the head, where some specimens had paler margins on the anterior portion of the labrum and the lateral margins of the clypeus, most were uniformly black.

##### Etymology.

The species name is derived from the Latin *flavus*, meaning “yellow”, after the distinct yellow margins of the pronotum and elytra.

##### Distribution.

Known from the Andean States of Táchira and Barinas (Fig. 17).

##### Biology.

A long series of this species was collected in a small pool that was formed by a road-cut in the Andes (Fig. 19D). It was being fed by a small stream/seepage, in which two additional specimens were collected. The specimen from Táchira was found in a small pool along a dirt road.

#### Chasmogenus gato sp. nov.

Taxon classificationAnimaliaColeopteraHydrophilidae

2D8F5681-4CD0-5BBF-8AC4-75B5201C5F9D

http://zoobank.org/E80A3658-638D-40CA-A2D5-D01BA0382CE2

Figures 9F , 14C , 17 , 20A

##### Type material.

**Holotype (male)**: “Venezuela: Amazonas State/ 4°58.838'N, 67°44.341'W; 95m/ Communidad Caño Gato on Rio/ Sipapo; 16.i.2009; leg. Short/ Miller, Camacho, Joly, & García/ VZ09-0116-01X: along stream”, “[barcode]/ SM0843374/ KUNHM-ENT”, “HOLOTYPE/ CHASMOGENUS/ gato sp. n./ des. Smith & Short” (MIZA). **Paratypes (19): Venezuela: Amazonas**: same data as holotype (14 exs., MIZA, SEMC, including DNA voucher SLE1202); River near Orinoco/Sipapo confluence, 5°03.707'N, 67°46.768'W, 92 m, 15.i.2009; leg K. Miller, detrital pools, VZ09-0115-01B (3 exs., SEMC); S. Communidad Porvenir, 5°20.514'N, 67°45.315'W, 87 m, 15.i.2009, leg. Short & García, pool in culvert, VZ09-0115-03A (1 ex., SEMC); ca. 15 km E. Puerto Ayacucho, 5°34.408'N, 67°30.283'W, 66 m, 6.i.2006, leg. A. E. Z. Short, stream at road crossing, AS-06-015 (1 ex., SEMC).

##### Differential diagnosis.

Of smaller species with a broadly rounded clypeal emargination, this species is similar in size, morphology, and general form of the aedeagus to *C. clavijoi* but can be differentiated by the slightly darker dorsal coloration, which is very dark brown.

##### Description.

***Size and color.*** Total body length 3.3–3.8 mm. Body form elongate oval with slightly curved lateral margins. Dorsum of head bicolored, frons and clypeus pale to dark brown, labrum pale yellow-brown (Fig. 9F). Pronotum and elytra dark orange-brown to dark brown. ***Head.*** Ground punctation on head fine, slightly finer on labrum. Clypeus with anteromedial emargination, which exposes a rounded to angulate gap between the labrum and clypeus (Fig. 9F). Mentum moderately depressed in anterior half with subtriangular notch. Maxillary palps long, longer than width of head immediately posterior to eyes. ***Thorax.*** Ground punctation on pronotum fine. Prosternum very weakly tectiform. Mesoventrite with weak elevation forming a posteromedial longitudinal carina. Metafemora densely pubescent in basal nine-tenths. ***Aedeagus.*** Aedeagus (Fig. 14C) with median lobe widest at base and very slightly tapering until apical fifth, then narrowing to form an acute triangular apex which is even with the apex of the parameres. Sclerite of the median lobe not expanded. Gonopore situated less than half of one gonopore width below the apex of the median lobe. Parameres symmetrical, with outer margins straight, with apex slightly inwardly curved and bluntly rounded. Basal piece long, ca. four-fifths the length of the parameres.

##### Etymology.

The species is named after the community of Caño Gato, from where the species was collected. To be treated as a noun in apposition.

##### Distribution.

Known from several closely situated localities in southern Venezuela (Amazonas) (Fig. 17).

##### Biology.

This species has been collected in stream margins and associated stream habitats such as nearby detrital pools (Fig. 20A).

#### Chasmogenus guianensis sp. nov.

Taxon classificationAnimaliaColeopteraHydrophilidae

73A338D4-5D3D-57EC-AE9A-C2104AA961DC

http://zoobank.org/F43EBB30-B396-4945-9AF0-31563B4F3087

Figures 10C , 14G, H , 17 , 21F , 22C

Chasmogenus sp. X Short, 2013: 87 (in part); Short & Kadosoe, 2011: 87 (in part).

##### Type material.

**Holotype (male)**: “Suriname: Sipaliwini District/ 2.47700N, 55.62941W, 275 m/ Camp 1, Upper Palumeu/ leg. A. Short; large sandy creek/ 14.iii.2012; SR12-0314-01A/2012 CI-RAP Survey”, “[barcode]/SEMC1088252/KUNHM-ENT”, “HOLOTYPE/ CHASMOGENUS/ guianensis sp. n./ des. Smith & Short.” (NZCS). **Paratypes (235): Suriname: Para District**: near Overbridge River Resort, 05°31.8'N, 055°03.5'W, 15-18-FEB-2010, Flight Intercept Trap, leg. P. Skelley, W. Warner, and C. Gillett (1 ex., SEMC). **Sipaliwini District**: same data as holotype (55 exs., NZCS, SEMC, including DNA Voucher SLE1826), same data as holotype except: 10.iii.2012, small forest pool, SR12-0310-02A (27 exs., SEMC); same data as holotype except: 10–12.iii.2012, large detrital pools, SR12-0310-01A (8 exs., SEMC); same data as holotype except: 11.iii.2012, large pool by trail, SR12-0311-01A (73 exs., SEMC, including DNA Vouchers SLE1827 and SLE1863); same data as holotype except: water held in dead palm leaf, SR12-0311-02A (1 ex., SEMC); same data as holotype except: 10–16.iii.2012, Flight Intercept Trap, SR12-0310-TN1 (44 exs., SEMC); Rapids on Kutari River, 2°19.280'N, 56°52.595'W, 224 m, 18.viii.2010, leg. A. E. Z. Short, 2010 CI-RAP Survey, forest stream, SR10-0818-01A (1 ex., SEMC); Camp 2 on Sipaliwini River 2°10.973'N, 56°47.235'W; 210 m, 28–29.viii.2010, leg. Short and Kadosoe 2010 CI-RAP Survey, large forest stream, SR10-0828-02A (1 ex., SEMC, DNA Voucher SLE1821); same data as previous except: 28.viii.2010, small detrital stream, SR10-0828-03A (1 ex., SEMC). **Guyana: Region 6**: Upper Berbice Basecamp 1, 4°09.289'N, 58°10.717'W, 96 m, 24.xi.2014, leg. Short, Salisbury, and La Cruz, margins of basecamp creek, GY14-0924-01A (15 exs., CBDG, SEMC, including DNA Vouchers SLE1616, SLE1836, and SLE1862); same data as previous except: 21.xi.2014, muddy detrital pools in drying creek bed near camp, GY14-0921-02A (5 exs., SEMC, including DNA Vouchers SLE1834 and SLE1835); Upper Berbice ca. 1 km south of Basecamp 1, 4°09.241'N, 58°10.627'W, 109 m, 25.ix.2014, leg. Short, Salisbury, and La Cruz, detritus pools in dry creek bed, GY14-0925-01D (3 exs., SEMC).

##### Differential diagnosis.

Among species that have a broad, rounded clypeal emargination, this species belongs to a group that have an aedeagal form with relatively broad, straight parameres and a wide median lobe which is slightly shorter to slightly longer than the apex of the parameres. It is distinctly smaller than *C. amplius*, *C. berbicensis*, and *C. clavijoi*. It is most similar to the comparably-sized *C. brownsbergensis* but the parameres are slightly wider and sublinear along the outer margins (Fig. 14G, H). Examination of the aedeagus is the only way to definitively identify this species. Unassociated females may not be determined with certainty.

##### Description.

***Size and color.*** Total body length 3.6–4.0 mm. Body form elongate oval with slightly curved lateral margins. Dorsum of head bicolored, frons dark orange-brown, clypeus and labrum dark orange to dark yellow-orange (Fig. 10C). Pronotum and elytra dark orange-brown. Venter dark red-brown mesally, dark brown marginally. ***Head.*** Ground punctation on head moderately coarse. Clypeus with anteroposterior emargination, which exposes a rounded to angulate gap between clypeus and labrum (Fig. 10C). Mentum moderately depressed in anterior half with rounded notch on anteromedial margin. Maxillary palps long, longer than width of head immediately posterior to eyes. ***Thorax.*** Ground punctation on pronotum moderately coarse. Prosternum even, not tectiform. Mesoventrite with very weak elevation forming a thin posteromedial longitudinal carina. Metafemora densely pubescent in basal nine-tenths. ***Aedeagus.*** Aedeagus (Fig. 14G, H) with median lobe widest at base and very slightly tapering along entire length, terminating in a bluntly triangular apex which is distinctly shorter than the apex of the parameres. Sclerite of the median lobe not expanded. Gonopore situated nearly one gonopore width below the apex of the median lobe. Parameres symmetrical, with outer margins straight, with apex slightly inwardly curved and bluntly rounded. Basal piece long, ca. three-quarters the length of the parameres.

##### Etymology.

This species is named after the Guiana Shield, the region of South America from which it is known. To be treated as a noun in apposition.

##### Distribution.

This species has been found at several localities in Guyana and Suriname (Fig. 17).

##### Biology.

This species was found in a variety of habitats including a forested swamp, ephemeral detrital pools, general creek margins and a large sandy creek (Fig. 21F). It was also collected passively via a flight intercept trap. A single specimen was collected from water in a dead palm leaf on the ground.

#### Chasmogenus ignotus sp. nov.

Taxon classificationAnimaliaColeopteraHydrophilidae

D0397F55-EFA4-546E-A48F-A055D5A9314C

http://zoobank.org/8DCFAD4B-4C3E-45C6-AAB9-1D92CD9488EC

Figures 16E–H , 17

##### Type material.

**Holotype (male)**: “Brazil: Amazonas: Manaus/ -2.93079, -59.97514, 75 m/ Ducke Reserve, near Station/ 5–10.vi.2018; leg. Short & Team/ Flight Intercept Trap/ BR18-0607-FIT”, “HOLOTYPE/ CHASMOGENUS/ ignotus sp. n./ des. Smith & Short” (INPA). **Paratypes (6): Brazil: Amazonas**: Same data as holotype (5 exs., INPA, SEMC); same locality but trail to Igarape Barro Branco, 6.vi.2018, leg. Short & Team, isolated forest pools, BR18-0606-02A (1 ex., SEMC DNA voucher SLE-1844).

**Figure 16.**
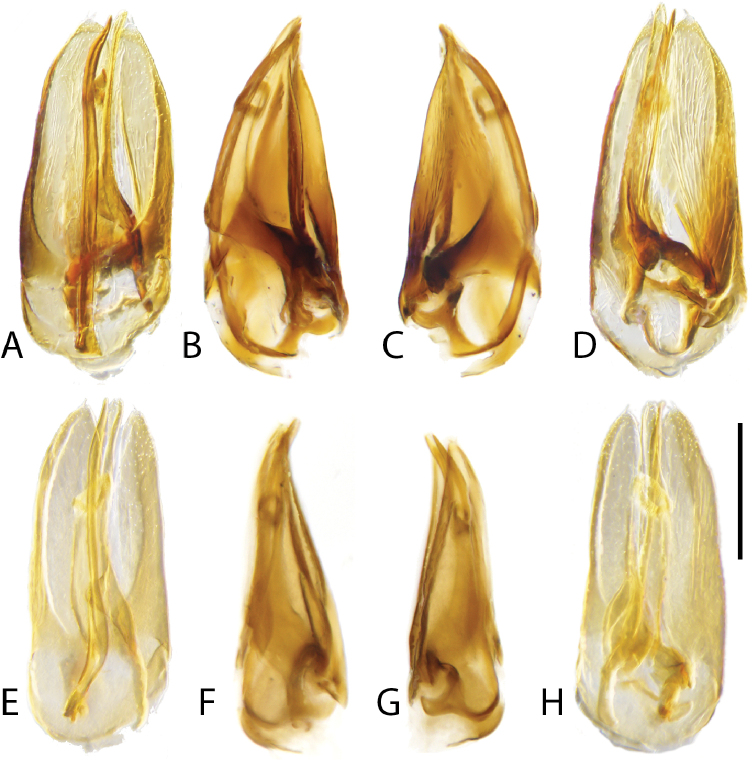
Aedeagi of *Chasmogenus* spp. **A–D***C. tafelbergensis***A** dorsal view **B** side view **C** side view **D** ventral view **E–H***C. ignotus***E** dorsal view **F** side view **G** side view **H** ventral view. Scale bar: 0.25 mm.

##### Differential diagnosis.

The strongly asymmetrical parameres, highly reduced basal piece, and extraordinary depth of the aedeagus (Fig. 16E–H) will easily separate this species from all others except *C. tafelbergensis*, from which it may be separated by its wider aedeagal profile. Examination of the aedeagus is the only way to definitively identify this species. Unassociated females may not be determined with certainty.

##### Description.

***Size and color.*** Total body length 3.5 mm. Dorsum of head, pronotum and elytra uniformly dark brown. Venter slightly lighter brown. ***Head.*** Ground punctation on head fine. Clypeus with anteroposterior emargination which exposes a very narrow, broad gap between clypeus and labrum. Mentum strongly depressed in anterior two-thirds with triangular anteromedial notch. Maxillary palps long, as long as width of head immediately posterior to eyes. ***Thorax.*** Ground punctation on pronotum moderately coarse. Surface of prosternum even, not tectiform. Mesoventrite with weak elevation forming a posteromedial longitudinal carina. Metafemora densely and uniformly pubescent in basal nine-tenths. ***Aedeagus.*** Aedeagus (Fig. 16E–H) with median lobe highly modified, appearing as a narrow subparallel-sided strap and partly rotated laterally, with apex extending to the apex of the parameres to slightly beyond. Sclerite of the median lobe extremely well developed and appearing as a thin curved strut that extends the full length of the genitalia. Gonopore oriented laterally (Fig. 16F); situated more than twice gonopore width below the apex of the median lobe. Parameres asymmetrical, with left paramere wider than right paramere; outer margins of parameres strongly sclerotized, with the sclerotized region on the right paramere thicker than the left. Aedeagus, especially the parameres, thickened, such that it takes on a three-dimensional appearance (Fig. 16F, G); in lateral view widest at base with dorsal surface flat with ventral surface graduating tapering to the apex. Basal piece short, less than half the length of the parameres; partly obscured by the strongly sclerotized and enlarged base of the median lobe and parameres.

##### Etymology.

The species name is derived from the Latin *ignotus*, meaning “strange”, after the bizarre form of the aedeagus.

##### Distribution.

Only known from the Adolpho Ducke Forest Reserve in Manaus, Brazil.

##### Biology.

One specimen was collected in an isolated forest detrital pool near Igarape Barro Branco, while another series of specimens came from a flight intercept trap in the forest.

**Figure 17.**
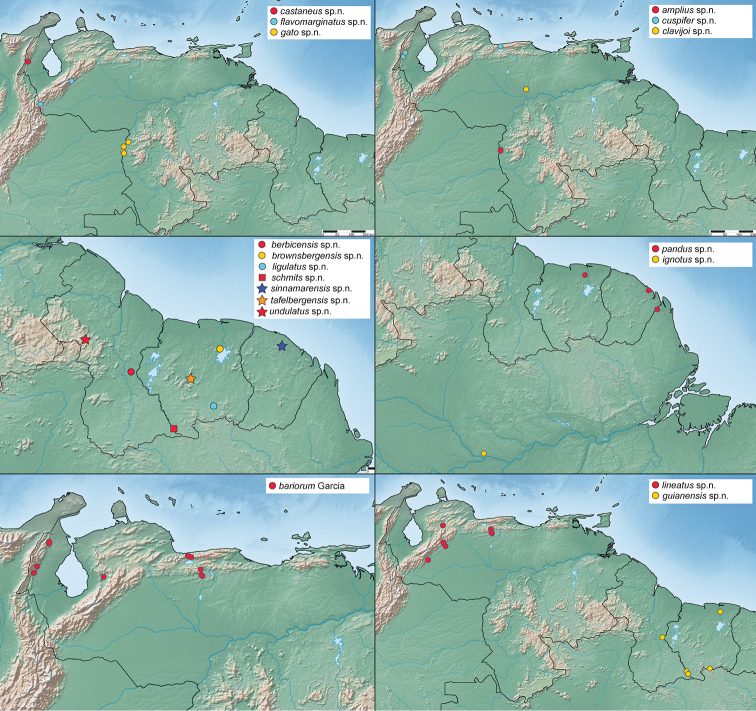
Distribution of *Chasmogenus* spp.

**Figure 18.**
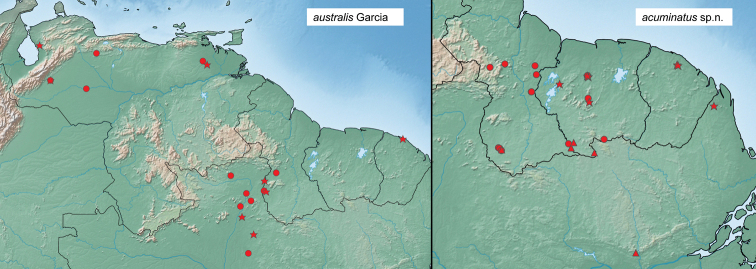
Distribution of *Chasmogenus* spp. Localities marked with stars/triangles indicate populations that were sampled for DNA in Fig. 1.

#### Chasmogenus ligulatus sp. nov.

Taxon classificationAnimaliaColeopteraHydrophilidae

220F1134-F685-576E-B449-C842D3699397

http://zoobank.org/F863DFA5-50EB-43C5-86DD-0F05DDD797C8

Figures 10B , 15E , 17 , 21B

Chasmogenus sp. X Short, 2013: 87 (in part).

##### Type material.

**Holotype (male)**: “Suriname: Sipaliwini District/ 2.97731N, 55.38500W, 200 m/ Camp 4 (low), Kasikasima; sandy/ creek, trail to Kasikasima; leg. A. Short/ 22.iii.2012; SR12-0322-02A/ flotation; 2012 CI-RAP Survey”, “[barcode]/ SEMC1086819/ KUNHM-ENT”. “HOLOTYPE/ CHASMOGENUS/ ligulatus sp. n./ des. Smith & Short” (NZCS). **Paratypes (5): Suriname: Sipaliwini**: same data as holotype except: 20.iii.2012, sandy stream on trail to METS camp, SR12-0320-02A (4 exs., SEMC, including DNA Voucher SLE474); same data as holotype except: 20–25.iii.2012, detrital pools along trail to METS camp, SR12-0320-03A (1 ex., SEMC).

##### Differential diagnosis.

The very thin gap between the clypeus and labrum (Fig. 10B) distinguish this species from all other congeners except for *C. sinnamarensis*, both of which are similar in size and morphology, but *C. sinnamarensis* is translucent dark red in dorsal coloration, and *C. ligulatus* is opaque and dark brown dorsally.

##### Description.

***Size and color.*** Total body length 3.2–3.5 mm. Body form elongate oval with slightly curved lateral margins. Dorsum of head multi-colored, frons dark brown, clypeus slightly paler, labrum distinctly paler (Fig. 10C). Pronotum dark orange-brown, slightly paler marginally. Elytra uniformly dark orange-brown to dark brown. Venter orange-brown mesally, dark brown marginally. ***Head.*** Ground punctation on head fine to moderately dense. Clypeus with anteromedial emargination which exposes a shallowly rounded gap between the labrum and the clypeus (Fig. 10C). Mentum strongly depressed in anterior two-thirds with triangular notch. Maxillary palps long, longer than width of head immediately posterior to eyes. ***Thorax.*** Ground punctation on pronotum fine. Prosternum very weakly tectiform. Mesoventrite with weak elevation forming a posteromedial longitudinal carina. Metafemora densely and uniformly pubescent in basal nine-tenths. ***Aedeagus.*** Aedeagus (Fig. 15E) with median lobe nearly parallel-sided but curved slightly to the left; apex bluntly rounded, distinctly extending beyond the apex of the parameres. Sclerite of the median lobe expanded and developed into a long, narrow crescent. Gonopore situated near the base of the median lobe. Parameres symmetrical, with outer margins strongly sinuate; the basal half of the parameres parallel sided with apical half appearing greatly expanded, then tapering to a blunt apex. Basal piece short, ca. one-third the length of the parameres.

##### Etymology.

The species name is derived from the Latin *ligula*, meaning “tongue” after the broad and extended tongue-like form of the median lobe of the aedeagus.

##### Distribution.

Known from lowland rainforest near the base of Mt. Kasikasima in southern Suriname (Fig. 17).

##### Biology.

Two of the three collecting events for this species were from small sandy streams in dense rainforest (Fig. 21B). A single specimen was also collected from a very large nearby detrital pool that was draining into a stream.

#### Chasmogenus lineatus sp. nov.

Taxon classificationAnimaliaColeopteraHydrophilidae

5C1D8F36-2A87-5983-8441-AD89F2C949E5

http://zoobank.org/78AFBB94-2494-4733-A4F8-B0CFBA090C98

**Figure 19.**
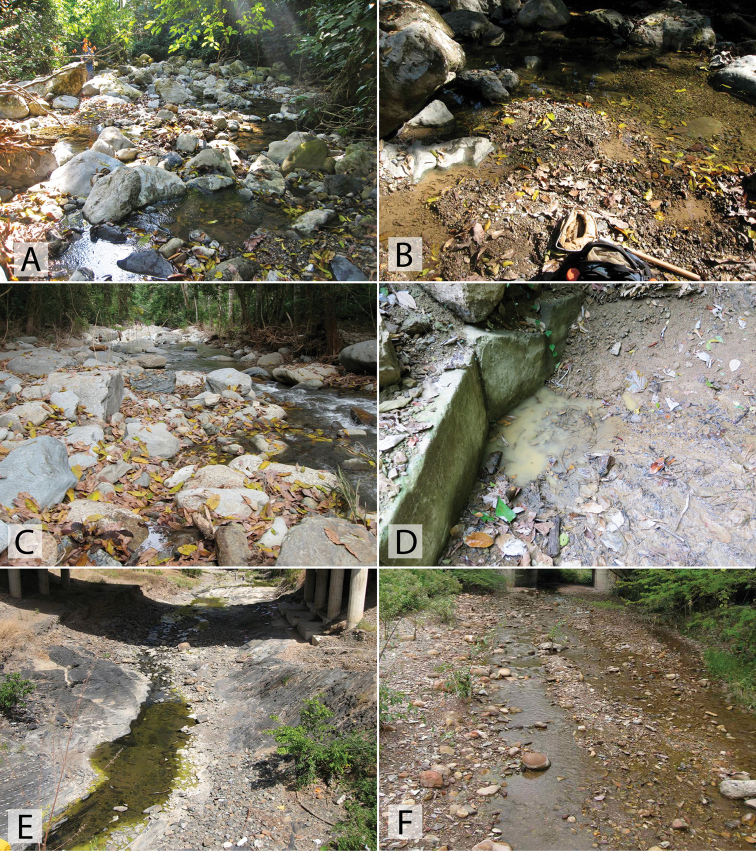
Habitat of *Chasmogenus* spp in the Andean region of Venezuela. **A, B** Type locality and habitat for *C. bariorum*, *C. cuspifer*, and *C. castaneus*, near El Tukuko, Rio Manantial collecting event VZ09-0129-01A) **C** habitat for *C. bariorum* and *C. cuspifer*, Henri Pittier National Park, Rio Cumboto (collecting event VZ09-0104-02B) **D** type locality and habitat for *C. flavomarginatus*, ca. 13 km NW of Baranitas (collecting event VZ12-0124-02B) **E** habitat for *C. bariorum* and *C. lineatus*, between San Juan and Dos Caminos, Rio San Antonio (collecting event VZ09-0108-02A) **F** habitat for *C. bariorum* and *C. lineatus*, near Arenales, Rio Salado (collecting event VZ09-0122-01X).

Figures 4A–C , 8F , 12E–I , 17 , 19E, F

##### Type material.

**Holotype (male)**: “Venezuela: Guárico State/ 9°46.320'N, 67°21.177'W, 280m/ Río San Antonio, N. Dos Caminos/ 8.i.2009; leg. Short, Miller & García/ VZ09-0108-02A: river margins”, “[barcode]/ SEMC0864029/ KUNHM-ENT”, “HOLOTYPE/ CHASMOGENUS/ lineatus sp. n./ des. Smith & Short” (MIZA). **Paratypes (247): Venezuela: Barinas**: River near Bum Bum, 8°18.033'N, 70°45.201'W, 216 m, 15.vii.2009, leg. A. Short et al., river margins, VZ09-0715-02A (48 exs., MIZA, SEMC, including DNA voucher SLE 1772). **Guárico**: Same data as holotype (35 exs., SEMC); same data as holotype except: leg. Miller & Short, side stream, VZ09-0108-02B (3 exs., SEMC, including DNA voucher SLE1614); Río Guárico, north of San Juan, 9.95788N, 67.37773W, 435 m, 8.i.2009, leg, K. Miller and L. Joly, along river, VZ09-0108-01X (11 exs, SEMC); ~20 km north of Dos Caminos, 9°44.034'N, 67°19.00'W, 225 m, leg. A. E. Z. Short, gravelly margin of river, AS-06-020 (54 exs, SEMC); ~15 km south of San Juan, 9°46.321'N, 67°21.201'W, 255 m, 3.i.2006, leg. A. E. Z. Short, stream margin & rock pools, AS-06-005 (17 exs., SEMC). **Lara**: Rio Salado, west of Arenales, 10°9.260'N, 69°57.458'W, 490 m, 22.i.2009, leg. Short, García, and Camacho, gravel stream, VZ09-0122-01X (66 exs., MIZA, SEMC, including DNA voucher SLE1061). **Portuguesa**: Tributary of Rio Guanare, south of Biscucuy, 9°14.457'N, 69°55.994'W, 370 m, 19.i.2009, leg. Short, García, and Camacho, gravel stream, VZ09-0119-03X (8 exs., SEMC); Rio Guanare, north of Guanare, 8°25.773'N, 69°35.202'W, 185 m, 19.i.2009, leg. Short, García, and Camacho, main river, VZ09-0119-02A (4 exs., SEMC); Rio Are at Aparicion, 9°22.900'N, 69°23.153'W, 220 m, 22.i.2012, leg. Short & Arias, river margins, VZ12-0122-02A (1 ex., SEMC).

##### Differential diagnosis.

The lack of a clypeal emargination serves to distinguish *C. lineatus* from all other congeners except *C. flavomarginatus*, from which it may be separated by the almost completely black dorsal coloration of the head (Fig. 8F) and the absence of pale preocular patches (pale margins and preocular patches present in *C. flavomarginatus*).

##### Description.

***Size and color.*** Total body length 2.9–3.3 mm. Body form very elongate oval with straight, subparallel lateral margins. Dorsum of head very dark brown to black (Fig. 8F). Pronotum and elytra uniformly dark brown. Prosternum dark orange to dark brown. Mesoventrite uniformly dark orange to dark brown. Metaventrite dark brown, slightly paler on posterior margin. Trochanters and glabrous portion of femora red-orange. Abdominal ventrites dark brown, slightly paler mesally (Fig. 4C). ***Head.*** Ground punctation on head fine. Clypeus and labrum contiguous (Fig. 8F). Mentum very weakly depressed in anterior half with triangular notch, followed by a medial elevated curved ridge situated posterior to triangular notch. Maxillary palps long, longer than width of head immediately posterior to eyes. ***Thorax.*** Ground punctation on pronotum fine. Prosternum moderately tectiform. Mesoventrite with weak elevation forming a posteromedial longitudinal carina with its distolateral margins slightly convex. Metafemora densely pubescent in basal three-fourths (Fig. 4F). ***Aedeagus.*** Aedeagus (Fig. 12E–I) with median lobe subtriangular in shape, widest at base and gradually tapering along entire length; apex acute, which extends slightly beyond the apex of the parameres. Sclerite of the median lobe not expanded. Gonopore situated slightly more than two gonopore widths below the apex of the median lobe. Parameres symmetrical, with outer margins slightly curved, appearing weakly convex in basal three-quarters, then abruptly angled outwards; apex bifid, appearing “mitten shaped”, with outer lobe very large, rounded, and directed outwards, and inner lobe very small and slightly more acuminate. Basal piece long, ca. as long as the length of the parameres.

##### Variation.

In some specimens examined, the abdominal ventrites were somewhat more densely pubescent than described in the taxonomic treatment. The color of the abdominal ventrites is also slightly variable; in some specimens either the anterior or posterior margin of each ventrite is slightly paler.

##### Etymology.

The species name is derived from the Latin *lineatus*, meaning “linear”, after the straight parallel-sided body form and also for the linear, unemarginated anterior margin of the clypeus.

##### Distribution.

This species is known from lowland gravel streams in the Andean region of Venezuela (Fig. 17).

##### Biology.

This species can be found in abundance in gravel streams along the foothills of the Merida Andes, especially exposed areas of gravel and sand with algae (Fig. 19E, F).

**Figure 20.**
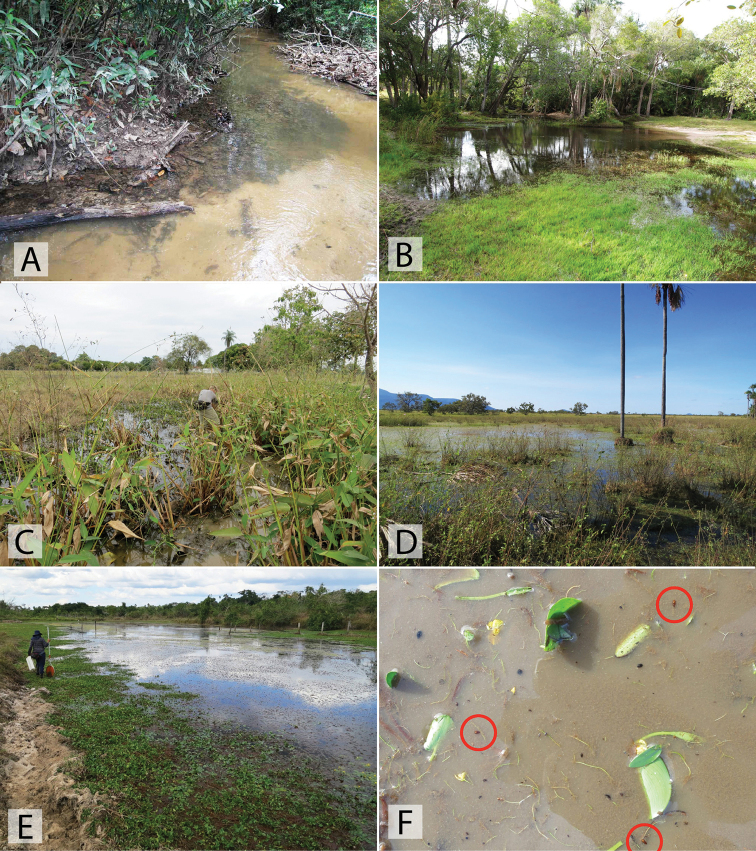
Habitat of *Chasmogenus* spp. **A** Type locality and habitat for *C. amplius* and *C. gato*, Venezuela: Amazonas, Caño Gato (collecting event VZ09-0116-01X) **B** type locality and habitat for *C. clavijoi*, Venezuela: Guarico, San Nicolasito Field Station (collecting event VZ09-0110-02X) **C–F** habitat for *C. australis*: **C** Venezuela, Barinas, 13 km southeast of Ciudad Bolivia (collecting event VZ12-0125-02A) **D** Guyana, near Kusad Mountains (collecting event GY13-1027-01A). **E, F** Brazil, Roraima, near Caracarai, red circles showing live individuals of *C. australis* floating on the water surface (collecting event BR18-0116-05A).

#### Chasmogenus pandus sp. nov.

Taxon classificationAnimaliaColeopteraHydrophilidae

77030676-C7F6-5C96-B0B7-C8A8C8AB0B1E

http://zoobank.org/C82E6A49-872D-45A7-A9D9-114A54E677D0

Figures 5D–F , 10D , 15A–D , 17

##### Type material.

**Holotype (male)**: “Suriname: Para District/ Zanderij, near Guesthouse/ 05°27.5'N, 055°13.0'W/ 9-18-FEB-2010; P.Skelley,/ W.Warner, C.Gillett; FIT”, ”[barcode]/SEMC1085915/ KUNHM-ENT”, “HOLOTYPE/ CHASMOGENUS/ pandus sp. n./ des. Smith & Short.” (NZCS). **Paratypes (15): Brazil: Amapa**: ca. 1 km E Oiapoque, 3.85039°, -51.81683°, 17 m, 18.vii.2018, leg. Short, flotation of detritus on forested seep, BR18-0718-03C (1 ex., INPA, DNA Voucher SLE1858). **French Guiana**: Roura, 27.4 km south-southeast, 4°44'20"N, 52°13'25"W, 280 m, 10 JUN 1997, leg. J. Ashe and R. Brooks, Flight Intercept Trap, FG1AB97 177 (2 exs., SEMC); same data as previous except: 23–24 May 1997, FG1AB97 022 (1 ex., SEMC); same data as previous except: 25–29 May 1997, FG1AB97 079 (1 ex., SEMC). **Suriname: Para District**: same data as holotype (9 exs., SEMC); same data as previous except: 7-9-FEB-2010, lights (1 ex., SEMC).

##### Differential diagnosis.

The very narrow gap between the clypeus and the labrum (Fig. 10D) is a characteristic shared only with *C. ligulatus*, but the two differ in the form of the aedeagus, in which the median lobe of *C. pandus* is very thin and the parameres are parallel-sided along the outer margins (Fig. 15A–D), which contrasts with the very convex parameres of *C. ligulatus*. Examination of the aedeagus is the only way to definitively identify this species. Unassociated females may not be determined with certainty.

##### Description.

***Size and color.*** Total body length 3.5–3.7 mm. Body form elongate oval with slightly curved lateral margins. Dorsum of head bicolored, frons dark red-brown, clypeus and labrum slightly paler (Fig. 10D). Pronotum and elytra uniformly dark brown. Venter uniformly dark brown (Fig. 5F). ***Head.*** Ground punctation on head moderately coarse. Clypeus with medial anteroposterior emargination which exposes a very narrow, wide gap between the clypeus and labrum (Fig. 10D). Mentum strongly depressed in anteromedial two-thirds with subtriangular notch. Maxillary palps long, longer than width of head immediately anterior to the eyes. ***Thorax.*** Ground punctation on pronotum fine. Prosternum tectiform. Mesoventrite with elevation forming a posteromedial longitudinal carina, increasing in elevation anteroposteriorly with highest elevation near protrochanters; slightly convex along outer margins. Metafemora densely pubescent in basal six-sevenths (Fig. 5F). ***Aedeagus.*** Aedeagus (Fig. 15A–D) with median lobe nearly parallel-sided and widest in basal half, then angled slightly to the left and tapering gradually to a weakly acuminate apex, distinctly extending beyond the apex of the parameres. Sclerite of the median lobe expanded and developed into a long, narrow sliver with a sharply acute apex that extends to the apex of the parameres. Gonopore situated in the middle of the median lobe, ca. two gonopore widths below the apex. Parameres symmetrical, with outer margins strongly bisinuated, giving the margins a weakly undulating appearance, apical half not wider than basal half; apex bluntly rounded. Basal piece short, ca. one-third the length of the parameres.

##### Etymology.

The species name is derived from the Latin *pandus*, meaning “bent” after the curved sclerite of the medial lobe of the aedeagus.

##### Distribution.

Known from Brazil (Amapá), Suriname (Para District), and French Guiana (Fig. 17).

##### Biology.

Specimens from French Guiana and Suriname were collected via a Flight Intercept Trap. The single specimen from Brazil was collected by floating detritus in a forested seepage.

##### Remarks.

Most examined specimens appeared to be more translucent than is typical of most species of *Chasmogenus*. It is unknown if this was due to preservation method or an actual diagnostic feature of this species.

#### Chasmogenus schmits sp. nov.

Taxon classificationAnimaliaColeopteraHydrophilidae

73228F3E-D885-5196-8CD5-78044BB1472C

http://zoobank.org/43FCAE8F-5A93-4E35-99C6-8E937EE27521

**Figure 21.**
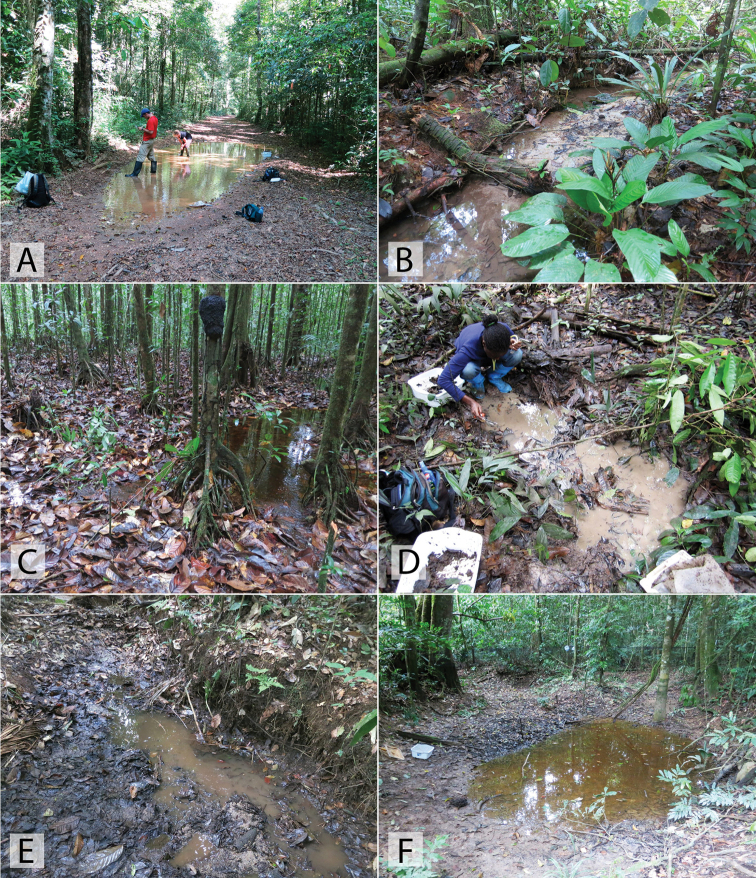
Habitat of *Chasmogenus* spp in Suriname. **A** Type locality and habitat for *C. brownsbergensis*, Brownsberg Nature Park (collecting event SR12-0804-03A) **B** type locality and habitat for *C. ligulatus*, near Mt. Kasikasima (collecting event SR12-0320-02A) **C** type locality and habitat for *C. tafelbergensis*, summit of Tafelberg tepui (collecting event SR13-0817-01A) **D** type locality and habitat for *C. schmits*, S of Kwamala along the Kutari River (collecting event SR10-0316-01B) **E** habitat for *C. acuminatus*, Central Suriname Nature Reserve, Raleighvallen (collecting event SR16-0316-01B) **F** habitat for *C. guianensis*, upper Palumeu River, (collecting event SR12-0311-01A).

Figures 14F , 17 , 21D

Chasmogenus sp. X Short & Kadosoe, 2011: 87 (in part).

##### Type material.

**Holotype (male)**: “Suriname: Sipaliwini District/ 2°10.521'N, 56°47.244'W, 228 m/ on Kutari River; leg. Short/ & Kadosoe; forested swamp/ 19.viii.2010; SR10-0819-01A/ Camp 1; 2010 CI-RAP Survey”/ “[barcode] SEMC0914251/KUNHM-ENT”, “HOLOTYPE/ CHASMOGENUS/ schmits sp. n./ des. Smith & Short”, “DNA VOUCHER/ Extraction #/ SLE-1824” (NZCS). **Paratypes (2): Suriname: Sipaliwini**: same data as holotype (2 exs., SEMC).

##### Differential diagnosis.

This species is similar morphologically to *C. clavijoi*, but the median lobe emarginates laterally to a greater degree and is only as wide as one paramere basally (Fig. 14F), whereas the median lobe is ca. 1.5× the width of one paramere basally in *C. clavijoi*. The apices of the parameres are also more squarely blunted rather than roundly curved. Examination of the aedeagus is the only way to definitively identify this species. Unassociated females may not be determined with certainty.

##### Description.

***Size and color.*** Total body length 4.3 mm. Body form elongate oval with slightly curved lateral margins. Dorsum of head dark brown, labrum distinctly paler. Pronotum and elytra uniformly dark brown. Venter dark orange-brown. ***Head.*** Ground punctation on head fine. Clypeus with anteromedial emargination which exposes trapezoidal gap between clypeus and labrum. Mentum moderately depressed in anterior half with triangular anteromedial notch. Maxillary palps long, longer than width of head immediately posterior to eyes. ***Thorax.*** Ground punctation on pronotum fine. Prosternum mildly tectiform. Mesoventrite with weak elevation forming a posteromedial longitudinal carina. Metafemora densely and uniformly pubescent in basal nine-tenths. ***Aedeagus.*** Aedeagus (Fig. 14F) with median lobe widest at base, appearing weakly constricted medially, then narrowing abruptly in the apical fifth to form an acute triangular apex which is slightly below the apex of the parameres. Sclerite of the median lobe not expanded. Gonopore situated less than a third of one gonopore width below the apex of the median lobe. Parameres symmetrical, with outer margins straight, with apex slightly inwardly curved and bluntly rounded. Basal piece long, ca. four-fifths the length of the parameres.

##### Etymology.

Named in honor of Sarah C. Schmits, a longtime member of the Short Lab who has provided invaluable support to advance and disseminate knowledge of aquatic beetles.

##### Distribution.

Known only from the type locality in Suriname (Fig. 17).

##### Biology.

This species was collected from a forested swamp habitat (Fig. 21D).

#### Chasmogenus sinnamarensis sp. nov.

Taxon classificationAnimaliaColeopteraHydrophilidae

E394B9E1-53C3-5218-B5D7-2E0B1C326C64

http://zoobank.org/DF6731D8-AD00-498E-B2B1-083827DACCB0

Figures 15F , 17

##### Type material.

**Holotype (male)**: “French Guyana:/ Road Petit Saut,/ Crique Eau Claire/ xii.2002–1.2003./ M. Balke leg.” , “DNA VOUCHER/ Extraction #/ SLE-77” (SEMC). **Paratypes (3)**: same data as holotype (3 exs., SEMC including DNA Voucher SLE517).

##### Differential diagnosis.

*Chasmogenus sinnamarensis* is similar in morphology and size to *C. berbicensis*, but can be differentiated from that species by the form of the mentum, which possesses an elevated ridge posterior to the anteromedial notch. This mentum character is also shared with the Andean species *C. bariorum*, *C. cuspifer*, and *C. lineatus*, however these species either have a distinctly toothed mesoventrite (not toothed in *C. sinnamarensis*) or clypeus without an anteromedial emargination (emarginated in *C. sinnamarensis*).

##### Description.

***Size and color.*** Total body length 4.9–5.0 mm. Body form elongate oval with slightly curved lateral margins. Dorsum of head dark brown. Pronotum dark brown with slightly paler lateral margins, elytra uniformly dark brown. ***Head.*** Ground punctation on head fine. Clypeus with anteromedial emargination, which exposes broadly rounded shaped gap between clypeus and labrum. Mentum moderately depressed in anterior half with rounded anteromedial notch followed by rounded elevated ridge just posterior of the notch. Maxillary palps long, longer than width of head immediately posterior to eyes. ***Thorax.*** Ground punctation on pronotum fine. Prosternum mildly tectiform. Mesoventrite with very weak elevation forming a posteromedial longitudinal carina. Metafemora densely and uniformly pubescent in basal nine-tenths. ***Aedeagus.*** Aedeagus (Fig. 15F) with median lobe widest at base, nearly parallel sided in basal three-quarters, then narrowing abruptly to form an acute triangular apex which is even with or slightly extends beyond the apex of the parameres. Sclerite of the median lobe not expanded. Gonopore situated less than half of one gonopore width below the apex of the median lobe. Parameres symmetrical, with outer margins straight, with apex very slightly inwardly curved and bluntly rounded. Basal piece long, ca. as long as the length of the parameres.

##### Etymology.

Named after the Sinnamary River, close to where it was collected. To be treated as a noun in apposition.

##### Distribution.

Only known from the type locality in French Guiana (Fig. 17).

##### Biology.

This species was collected from a clear water creek.

#### Chasmogenus tafelbergensis sp. nov.

Taxon classificationAnimaliaColeopteraHydrophilidae

A3EBDEBF-036E-5B48-83DE-5310733DB7B7

http://zoobank.org/D3640B54-0826-4469-A091-AF1164BBEA14

Figures 16A–D , 17 , 21C

##### Type material.

**Holotype (male).** “Suriname: Sipaliwini District/ 3°55.600'N 56°11.300'W, 600m/ CSNR: Tafelberg Summit, nr/ Augustus Creek Camp, pools &/ creeks on trail into Arrowhead/ basin; leg. Short & Bloom. 17.viii.2013; SR13-0817-01A”, “HOLOTYPE/ CHASMOGENUS/ tafelbergensis sp. n./ des. Smith & Short”, “DNA VOUCHER/ Extraction #/ SLE-1825”. (NZCS).

##### Differential diagnosis.

The strongly asymmetrical parameres, highly reduced basal piece, and extraordinary depth of the aedeagus (Fig. 16A–D) will easily separate this species from all others except *C. ignotus*, from which it may be differentiated by its narrower aedeagal profile. Examination of the aedeagus is the only way to definitively identify this species. Unassociated females may not be determined with certainty.

##### Description.

***Size and color.*** Total body length 3.3 mm. Body form elongate oval with slightly curved lateral margins. Dorsum of head bicolored, frons dark red-brown, clypeus and labrum dark orange-brown. Pronotum and elytra uniformly dark brown. ***Head.*** Ground punctation on head moderately coarse. Clypeus with anteromedial emargination, which exposes angulate gap between clypeus and labrum. Mentum moderately depressed in anterior half with anteromedial subtriangular notch. Maxillary palps long, longer than width of head immediately posterior to eyes. ***Thorax.*** Ground punctation on pronotum moderately coarse. Prosternum mildly tectiform. Mesoventrite with very weak elevation forming a posteromedial longitudinal carina. Metafemora densely pubescent in basal six-sevenths. ***Aedeagus.*** Aedeagus (Fig. 16A–D) with median lobe highly modified, appearing as a narrow subparallel-sided strap and partly rotated laterally, with apex extending to the apex of the parameres. Sclerite of the median lobe extremely well developed and appearing as a thin curved strut that extends the full length of the genitalia. Gonopore oriented laterally (Fig. 15B); situated twice gonopore width below the apex of the median lobe. Parameres asymmetrical, with left paramere wider than right paramere; outer margins of parameres strongly sclerotized, with the sclerotized region on the right paramere thicker than the left. Aedeagus, especially the parameres, thickened, such that it takes on a highly three-dimensional appearance (Fig. 16B, C); in lateral view widest in basal half, with dorsal surface strongly convex, with ventral surface nearly flat. Basal piece short, less than half the length of the parameres; partly obscured by the strongly sclerotized and enlarged base of the median lobe and parameres.

##### Etymology.

Named after Tafelberg, a low elevation sandstone tepui in central Suriname. To be treated as a noun in apposition.

##### Distribution.

This species only known from the summit of Tafelberg Tepui in Suriname (Fig. 17).

##### Biology.

The single specimen of this species was collected from forested pools which contained extremely dense layers of leaf litter detritus (Fig. 21C).

#### Chasmogenus undulatus sp. nov.

Taxon classificationAnimaliaColeopteraHydrophilidae

9624C9B4-1A2D-5F29-9829-8842D6691D90

http://zoobank.org/8D86B749-C001-4FFB-8423-DDC75A7BA12B

**Figure 22.**
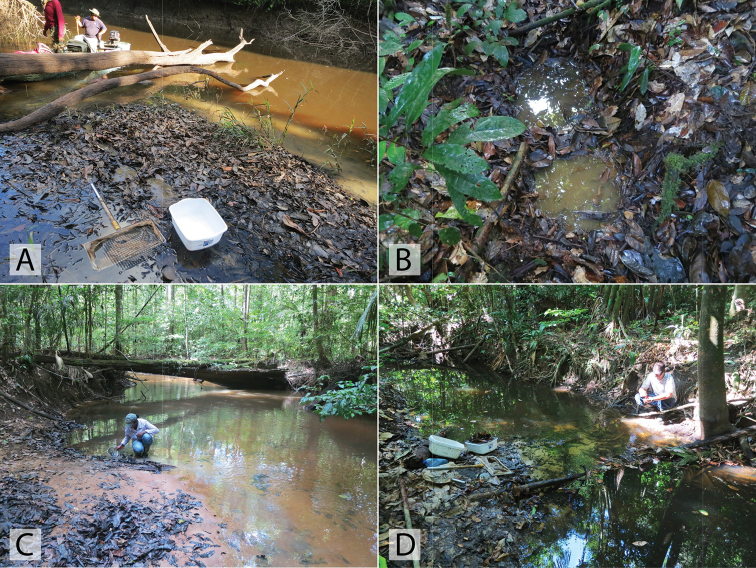
Habitat of *Chasmogenus* spp in Guyana. **A** Type locality and habitat for *C. berbicensis*, Guyana, margin/detrital sandbar of the upper Berbice River (collecting event GY14-0922-02A) **B** type locality and habitat for *C. undulatus* Guyana, near Ayanganna airstrip (collecting event GY14-0317-01A) **C** habitat for *C. acuminatus* and *C. guianensis*, Guyana, small tributary of the upper Berbice River (collecting event GY14-0924-01A) **D** habitat for *C. acuminatus* (collecting event GY13-1103-02A).

Figures 9E , 15G , 17 , 22B

Chasmogenus sp. A Short, Salisbury, & La Cruz 2018: 193.

##### Type material.

**Holotype (male)**: “Guyana: Region XIII/ 5°18.261'N, 59°50.257'W; 687 m/ Ayanganna Airstrip, trail from air-/ strip to Ayanganna; forested/ detrital pools; leg. A. Short/ 18.iii.2014/ GY14-0318-01B”, “[barcode]/SEMC1313817/ KUNHM-ENT”, “HOLOTYPE/ CHASMOGENUS/ undulatus sp. n./ des. Smith & Short” (CBDG). **Paratypes (20)**: GUYANA: Region 8: same data as holotype (4 exs., SEMC including DNA Voucher SLE1618); same data as holotype except: 17.iii.2014, forest detrital pools, GY14-0317-01A (14 exs., CBDG, SEMC including DNA Vouchers SLE1832 and SLE1833); same data as holotype except: 19.iii.2014, trail from airstrip to marshy mined area, GY14-0319-02A (1 ex., SEMC); Ayanganna Airstrip, trail from Blackwater Creek Camp to Potaro River, 5°17.823'N, 59°50.000'W, 684 m, 19.iii.2014, leg. A. Short, forest detrital pools, GY14-0319-01A (1 ex., SEMC, DNA Voucher SLE1831).

##### Differential diagnosis.

Among species that have a broad clypeal emargination and the apex of the median lobe extending past the apex of the paramere, *C. undulatus* may be distinguished from the widespread and similar *C. acuminatus* by the distinctly sinuated outer margins of the parameres (Fig. 15G) (straight in *C. acuminatus*). In other species with sinuated outer margins of the parameres (*C. ligulatus* and *C. pandus*), this species may be distinguished by the gradually tapered and blunt apex of the median lobe (more pointed in *C. pandus*, not tapered in *C ligulatus*). Examination of the aedeagus is the only way to definitively identify this species. Unassociated females may not be determined with certainty.

##### Description.

***Size and color.*** Total body length 3.8–4.0 mm. Body form elongate oval with slightly curved lateral margins. Dorsum of head dark brown, clypeus slightly paler (Fig. 9E). Pronotum dark orange-brown, distinctly paler marginally. Elytra uniformly dark brown. Venter dark red brown to dark brown. ***Head.*** Ground punctation on head fine to moderately coarse. Clypeus with anteromedial emargination, which exposes a trapezoidal gap between the clypeus and labrum (Fig. 9E). Mentum strongly depressed in anterior half with subtriangular anteromedial notch. Maxillary palps long, longer than width of head immediately posterior to eyes. ***Thorax.*** Ground punctation on pronotum moderately coarse. Prosternum tectiform. Mesoventrite with elevation forming a posteromedial longitudinal carina with the lateral margins convex. Metafemora densely pubescent in basal six-sevenths. ***Aedeagus.*** Aedeagus (Fig. 15G) with median lobe nearly parallel-sided and widest in basal half, then angled slightly to the left and tapering gradually to a weakly acuminate apex, distinctly extending beyond the apex of the parameres. Sclerite of the median lobe expanded and developed into a wide angulate sliver with a sharply acute apex that extends to the apex of the parameres. Gonopore situated ca. 1.5 gonopore widths below the apex of the median lobe. Parameres symmetrical, with outer margins strongly bisinuated, giving the margins a weakly undulating appearance, apical distinctly narrower than basal half; apex bluntly rounded. Basal piece short, ca. one-third the length of the parameres.

##### Etymology.

The species name is derived from the Latin *undulatus*, meaning “wavy”, after the curvy and sinuated margins of the parameres.

##### Distribution.

This species is known from several collections near Mount Ayanganna in western Guyana.

##### Biology.

Series of this species were collected in forested detrital pools (Fig. 22B).

#### Chasmogenus

Taxon classificationAnimaliaColeopteraHydrophilidae

sp. C

2045C95A-D4F1-5109-8C93-8217F213E5CA

Chasmogenus sp. C Short, Salisbury, & La Cruz 2018: 193.

##### Material examined (4).

**Guyana: Region 6**: Upper Berbice, Basecamp 2, 4°45.301'N, 58°00.404'W, 49 m, 26.ix.2014, leg. Short, Salisbury and La Cruz, shallow detrital pools in forest draining into creek, GY14-0926-01A (4 females, CBDG, SEMC including DNA Voucher SLE1783).

##### Remarks.

This species is only known from four female specimens. We sequenced one specimen which was genetically distinct from all other described species in the region and likely represents an undescribed species. However, because the aedeagus is critical for identification, we have chosen not to formally describe this species until males can be found. This species is morphologically similar to *C. pandus*, particularly the reddish dorsal coloration and smaller size. It was first recognized as a distinct morphospecies by Short et al. (2018), where it was listed as “*Chasmogenus* sp. C”, and we have followed this naming convention for continuity.

### Key to species of *Chasmogenus* Sharp of Venezuela, Suriname, Guyana, French Guiana, and Brazil north of the Amazon River

**Table:** 

1	Anterior margin of clypeus straight, not emarginated (Figs 8E, F)	**2**
–	Anterior margin of clypeus emarginated, which may be narrow to broad (e.g., Figs 8A–D, 9A–F)	**3**
2	Dorsum of head completely black (Fig. 8F). Lateral margins of pronotum not distinctly paler than on disc (Fig. 4A)	***C. lineatus* sp. nov.**
–	Dorsum of head with pale preocular patches on the lateral margins of the clypeus (Fig. 8E). Lateral margins of pronotum distinctly paler than disc (Fig. 3A)	***C. flavomarginatus* sp. nov.**
3	Anterior emargination of the clypeus triangular (e.g., Fig. 8A–D). Andean region	**4**
–	Anterior emargination of clypeus broad and rounded (e.g. Figs 9A–F, 10A–D). Widespread but generally not from the Andean region (except the lowland *C. australis*)	**6**
4	Mesoventrite with longitudinal carinae that is strongly elevated into an acute tooth (Fig. 7A, B). Dorsal coloration dark brown to black (e.g. Fig. 3D)	**5**
–	Mesoventrite with longitudinal carinae, but never elevated into a tooth. Dorsal coloration dark reddish brown (Fig. 3G)	***C. castaneus* sp. nov.**
5	Body length > 3.5 mm; aedeagus with median lobe as wide as one paramere, tapering only at apical fourth (Fig. 11A–H)	***C. bariorum* García**
–	Body length < 3.5 mm; aedeagus with median lobe narrower than one paramere, tapering gradually and consistently along entire length (Fig. 12D)	***C. cuspifer* sp. nov.**
6	Body length < 4.5 mm, though typically less than 4.0 mm. Apex of median lobe of aedeagus ca. as long as the apex of the parameres (e.g., Figs 13A–D, 14A–E)	**10**
–	Body length > 4.5 mm. Apex of median lobe of aedeagus of variable length	**7**
7	Basal piece very long, subequal in length as length of parameres (Fig. 15F). Depression of mentum with anteromedial notch and anteroposteriorly curved rounded ridge posterior to notch	***C. sinnamarensis* sp. nov.**
–	Basal piece of aedeagus distinctly shorter than length of parameres (e.g. Fig. 14A–H). Depression of mentum without rounded ridge	**8**
8	Dorsal coloration usually dark brown. Venezuela and the Guianas	**9**
–	Dorsal coloration pale to medium brown (Fig. 2A). Aedeagus as in Fig. 14A.Venezuela	***C. amplius* sp. nov.**
9	Body length 4.5–4.9 mm. Aedeagus as in Fig. 14D. Guyana (Berbice River)	***C. berbicensis* sp. nov.**
–	Body length 3.8–4.5 mm. Aedeagus as in Fig. 14B. Venezuela (Rio Aguaro corridor)	***C. clavijoi* sp. nov.** (in part)
10	Body tan to very pale brown dorsal coloration (Fig. 4D). Typically found in open marsh/lentic habitats	***C. australis* García**
–	Body usually darker in overall coloration. Typically found in forested streams or forested detrital pools	**11**
11	Aedeagus highly asymmetrical, with parameres of unequal size and only partial sclerotization on dorsal surface; median lobe longitudinally divided and extremely narrow, basal piece oblique (Fig. 14B, C, 14F, G)	**12**
–	Aedeagus symmetrical or only slightly asymmetrical	**13**
12	Ventral face of aedeagus strongly curved, convex in lateral view (Fig. 16B)	***C. tafelbergensis* sp. nov.**
–	Ventral face of aedeagus nearly flat to slightly concave in lateral view (Fig. 16F)	***C. ignotus* sp. nov.**
13	Apex of median lobe long, distinctly extending beyond the apex of the parameres; basal piece short, one-third the total length of the parameres or less (e.g. Fig. 15A–E, 15G–I)	**14**
–	Apex of median lobe short, not extending beyond the apex of the parameres; basal piece long, up to one-half the length of the entire aedeagus	**17**
14	Parameres evenly curved along outer margins, not sinuate (Fig. 15H, I)	***C. acuminatus* sp. nov.**
–	Parameres slightly to strongly sinuate along outer margins (e.g. Fig. 15A, C–E)	**15**
15	Median lobe nearly uniform in width along entire length, with a broadly rounded apex (Fig. 15E)	***C. ligulatus* sp. nov.**
–	Median lobe gradually narrowing apically, with a narrowly rounded to pointed apex (Fig. 15A–D, G)	**16**
16	Median lobe with apex pointed (e.g., Fig. 15A–D); dorsal coloration dark reddish brown. Emargination of the clypeus extremely narrow and broad (Figs 5D–F, 10D)	***C. pandus* sp. nov.**
–	Median lobe with apex rounded (Fig. 15G); dorsal coloration dark brown. Emargination of the clypeus rounded to angulate	***C. undulatus* sp. nov.**
17	Apex of median lobe distinctly shorter than length of parameres (Fig. 14F, H)	**18**
–	Apex of median lobe approximately even with apex of parameres (Fig. 14B, C, E)	**19**
18	Apex of parameres bluntly rounded inwards, giving the apicomedial angle an almost toothlike appearance (Fig. 14F)	***C. schmits* sp. nov.**
–	Apex of parameres smoothly rounded inwards (Fig. 14G, H)...	***C. guianensis* sp. nov.**
19	Apex of parameres inwardly curved and distinctly narrowed along inner margin (Fig. 14E). Distinct dark patches on mesal portion of clypeus and labrum (Fig. 10A)	***C. brownsbergensis* sp. nov.**
–	Apex of parameres inwardly curved but not or only slightly narrowed along inner margin (Fig. 14B, C). Clypeus and labrum without distinct dark patches	**20**
20	Body length ≤ 3.8 mm, dorsal coloration darker, appearing very dark brown	***C. gato* sp. nov.**
–	Body length ≥ 3.8 mm, dorsal coloration paler, appearing orange-brown	***C. clavijoi* sp. nov.** (in part)

## Supplementary Material

XML Treatment for Chasmogenus

XML Treatment for Chasmogenus acuminatus

XML Treatment for Chasmogenus amplius

XML Treatment for Chasmogenus australis

XML Treatment for Chasmogenus bariorum

XML Treatment for Chasmogenus berbicensis

XML Treatment for Chasmogenus brownsbergensis

XML Treatment for Chasmogenus castaneus

XML Treatment for Chasmogenus clavijoi

XML Treatment for Chasmogenus cuspifer

XML Treatment for Chasmogenus flavomarginatus

XML Treatment for Chasmogenus gato

XML Treatment for Chasmogenus guianensis

XML Treatment for Chasmogenus ignotus

XML Treatment for Chasmogenus ligulatus

XML Treatment for Chasmogenus lineatus

XML Treatment for Chasmogenus pandus

XML Treatment for Chasmogenus schmits

XML Treatment for Chasmogenus sinnamarensis

XML Treatment for Chasmogenus tafelbergensis

XML Treatment for Chasmogenus undulatus

XML Treatment for Chasmogenus

## Supplementary materials

Supplementary material 1 Table S1. Pairwise uncorrected percentage similarity between *Chasmogenus*COI sequences used in this study Data type statistical data https://binary.pensoft.net/file/411778This dataset is made available under the Open Database License (http://opendatacommons.org/licenses/odbl/1.0/). The Open Database License (ODbL) is a license agreement intended to allow users to freely share, modify, and use this Dataset while maintaining this same freedom for others, provided that the original source and author(s) are credited.Rachel R. Smith, Andrew Edward Z. Short
